# Application of CRISPR-Cas12a temperature sensitivity for improved genome editing in rice, maize, and *Arabidopsis*

**DOI:** 10.1186/s12915-019-0629-5

**Published:** 2019-01-31

**Authors:** Aimee A. Malzahn, Xu Tang, Keunsub Lee, Qiurong Ren, Simon Sretenovic, Yingxiao Zhang, Hongqiao Chen, Minjeong Kang, Yu Bao, Xuelian Zheng, Kejun Deng, Tao Zhang, Valeria Salcedo, Kan Wang, Yong Zhang, Yiping Qi

**Affiliations:** 1 Department of Biotechnology, School of Life Science and Technology, Center for Informational Biology, University of Electronic Science and Technology of China, Chengdu, 610054 China; 2 Department of Plant Science and Landscape Architecture, University of Maryland, College Park, MD 20742 USA; 3 Crop Bioengineering Center, Iowa State University, Ames, Iowa 50011 USA; 4 Department of Agronomy, Iowa State University, Ames, Iowa 50011 USA; 5 Interdepartmental Plant Biology Major, Iowa State University, Ames, Iowa 50011 USA; 6 Jiangsu Key Laboratory of Crop Genetics and Physiology, Co-Innovation Center for Modern Production Technology of Grain Crops, Key Laboratory of Plant Functional Genomics of the Ministry of Education, Yangzhou University, Yangzhou, 225009 China; 7 Joint International Research Laboratory of Agriculture and Agri-Product Safety of Ministry of Education of China, Yangzhou University, Yangzhou, 225009 China; 8 Institute for Bioscience and Biotechnology Research, University of Maryland, Rockville, MD 20850 USA

**Keywords:** CRISPR-Cas12a, Temperature, Genome editing, Rice, *Arabidopsis*, Maize

## Abstract

**Background:**

CRISPR-Cas12a (formerly Cpf1) is an RNA-guided endonuclease with distinct features that have expanded genome editing capabilities. Cas12a-mediated genome editing is temperature sensitive in plants, but a lack of a comprehensive understanding on Cas12a temperature sensitivity in plant cells has hampered effective application of Cas12a nucleases in plant genome editing.

**Results:**

We compared AsCas12a, FnCas12a, and LbCas12a for their editing efficiencies and non-homologous end joining (NHEJ) repair profiles at four different temperatures in rice. We found that AsCas12a is more sensitive to temperature and that it requires a temperature of over 28 °C for high activity. Each Cas12a nuclease exhibited distinct indel mutation profiles which were not affected by temperatures. For the first time, we successfully applied AsCas12a for generating rice mutants with high frequencies up to 93% among T0 lines. We next pursued editing in the dicot model plant *Arabidopsis*, for which Cas12a-based genome editing has not been previously demonstrated. While LbCas12a barely showed any editing activity at 22 °C, its editing activity was rescued by growing the transgenic plants at 29 °C. With an early high-temperature treatment regime, we successfully achieved germline editing at the two target genes, GL2 and TT4, in *Arabidopsis* transgenic lines. We then used high-temperature treatment to improve Cas12a-mediated genome editing in maize. By growing LbCas12a T0 maize lines at 28 °C, we obtained Cas12a-edited mutants at frequencies up to 100% in the T1 generation. Finally, we demonstrated DNA binding of Cas12a was not abolished at lower temperatures by using a dCas12a-SRDX-based transcriptional repression system in *Arabidopsis*.

**Conclusion:**

Our study demonstrates the use of high-temperature regimes to achieve high editing efficiencies with Cas12a systems in rice, *Arabidopsis*, and maize and sheds light on the mechanism of temperature sensitivity for Cas12a in plants.

**Electronic supplementary material:**

The online version of this article (10.1186/s12915-019-0629-5) contains supplementary material, which is available to authorized users.

## Background

Many genome editing outcomes are achieved through sequence-specific nucleases (SSNs) which generate targeted DNA double-stranded breaks (DSBs). SSNs such as zinc-finger nuclease (ZFN) and transcription activator-like effector nuclease (TALEN) were widely applied in genome editing in plants [1]. However, in recent years, these SSNs have been overtaken by CRISPR (clustered regularly interspaced short palindromic repeats) systems with Cas9 or Cas12a (formerly Cpf1) nucleases that mediate DNA targeting through guide RNAs, which are easy to engineer [2–8].

CRISPR-Cas12a is an RNA-guided endonuclease that has provided new opportunities in genome editing through its distinct features from the commonly used CRISPR-Cas9 system. First, the PAM requirement for Cas12a is “TTTV” or “TTV,” which is advantageous for targeting promoters and other AT-rich sites in gene coding regions [3]. Cas12a requires only a ~ 43-nt guide CRISPR RNA (crRNA) and processes pre-crRNAs into mature RNAs, an ability that endows Cas12a systems with a natural multiplexing ability [9–12]. Additionally, Cas12a creates 4–5 bp staggered overhangs which may potentially facilitate gene replacement. Finally, Cas12a, unlike other nucleases such as Cas9, is not toxic in some organisms such as Chladymonas [13], which expands the spectrums of organisms that can benefit from genome editing. So far, three Cas12a varieties from different bacteria have been utilized for genome editing: *Francisella novicida* (FnCas12a), *Lachnospiraceae bacterium* (LbCas12a), and *Acidaminococcus* sp. *BV3L6* (AsCas12a), and all of them have been tested in plants [14–18].

Once a DSB is created by Cas12a or other type of endonucleases, it must be repaired through one of the two main repair pathways: non-homologous end joining (NHEJ) or homology-directed repair (HDR). NHEJ is the most commonly utilized pathway and often results in insertion/deletions (indels) that knock out targeted genes. Cas12a genome editing based on NHEJ has been demonstrated in a few plant species. In rice, editing was achieved at 12.1% mutation frequency using a small RNA promoter (OsU3) and a tRNA processing system to express crRNA [15]. By using a full-length repeat-spacer-repeat sequence and allowing LbCas12a’s endogenous processing system to create mature crRNA, mutation frequencies up to 41.2% were achieved in rice [16]. However, edited T0 plants were mostly heterozygous or chimeric and no homozygous plants were observed. We were able to achieve 100% mutation frequency in rice T0 plants using a double-ribozyme system with both Cas12a and crRNA under the control of the maize ubiquitin promoter (pZmUbi) [17]. In protoplasts, LbCas12a had an efficiency of 15–25%, while AsCas12a ranged from 0.6 to 10% [17]. The low editing efficiency of AsCas12a is consistent with previous results: one study could not detect any activity in rice T0 plants with AsCas12a and the other barely found AsCas12a-induced mutations in soybean protoplasts even when deep sequencing analysis was applied [15, 19]. Efficient maize genome editing up to 60% in T0 generation has also been achieved with LbCas12a [20]. In addition to LbCas12a, efficient editing has been shown by FnCas12a in rice and tobacco [12, 14]. Recently, we showed LbCas12a and FnCas12a were very specific for DNA targeting in plants [17, 18]. Using whole-genome sequencing, we could not detect any off-target mutations when targeting three sites in rice by LbCas12a [21], further demonstrating its high specificity.

In order to bolster Cas12a efficiency and expand its targeting scopes, two aspects were addressed respectively: crRNA design and PAM requirements. Because crRNAs for Cas12a are shorter than the single-guide RNAs for Cas9, undesired secondary structures noticeably decrease efficiency [22, 23] or even render the crRNA non-functional as we showed in maize [20]. Online tools CINDEL and CRISPR-DT can aid in crRNA design [22, 23]. Originally, FnCas12a was believed to have a PAM requirement of “TTV” [10]. However, this was questioned by two recent studies in human and rice cells [18, 24]. Lately, LbCas12a-RR and LbCas12a-RVR variants working at “CCCC,” “TYCV,” and “TATG” sites [25] were demonstrated in rice for expanded targeting scopes [18, 26].

Cas12a has genome editing applications beyond DSB generation; for example, Cas12a can recruit activators, repressors, or deaminases to the target site for either transcriptional regulation or base editing. We previously reported AsCas12a- and LbCas12a-based repressors that repressed *miR159b* in *Arabidopsis* to less than 10% of wild-type (WT) expression [17]. Interestingly, although AsCas12a was less efficient than LbCas12a in creating mutations, as a repressor, it was more consistent than LbCas12a in binding the targeted promoter to repress expression. While no reports have been published in plants, Cas12a-based transcriptional activators and base editors were successfully demonstrated in human cells [27, 28].

Cas12a nucleases and their engineered variants have been shown to efficiently and specifically cleave and bind DNA when crRNAs and processing systems are well designed. However, the pace of adoption of Cas12a systems to a wide collection of plant species has been slow, suggesting possible barriers for Cas12a technologies. One major barrier could be temperature as it was recently shown to affect Cas12a editing efficiency in zebrafish [29]. Interestingly, Cas9 editing was previously demonstrated to be impacted by temperature and higher temperatures helped achieve high editing efficiency in *Arabidopsis* and citrus [30]. Given plant transformation and growth are typically carried out at ambient temperatures (e.g., 20 to 25 °C), we reasoned that the difficulty of applying Cas12a nucleases in plants could be due to the fact that they are more temperature sensitive than Cas9. In this study, we systematically investigated this topic. Our results not only demonstrated that different Cas12a nucleases have differential activities and sensitivities to temperature in different plant species, but also shed light on the mechanism of temperature sensitivity for Cas12a.

## Results

### Temperature sensitivity of three Cas12a nucleases in rice cells

**Fig. 1:**
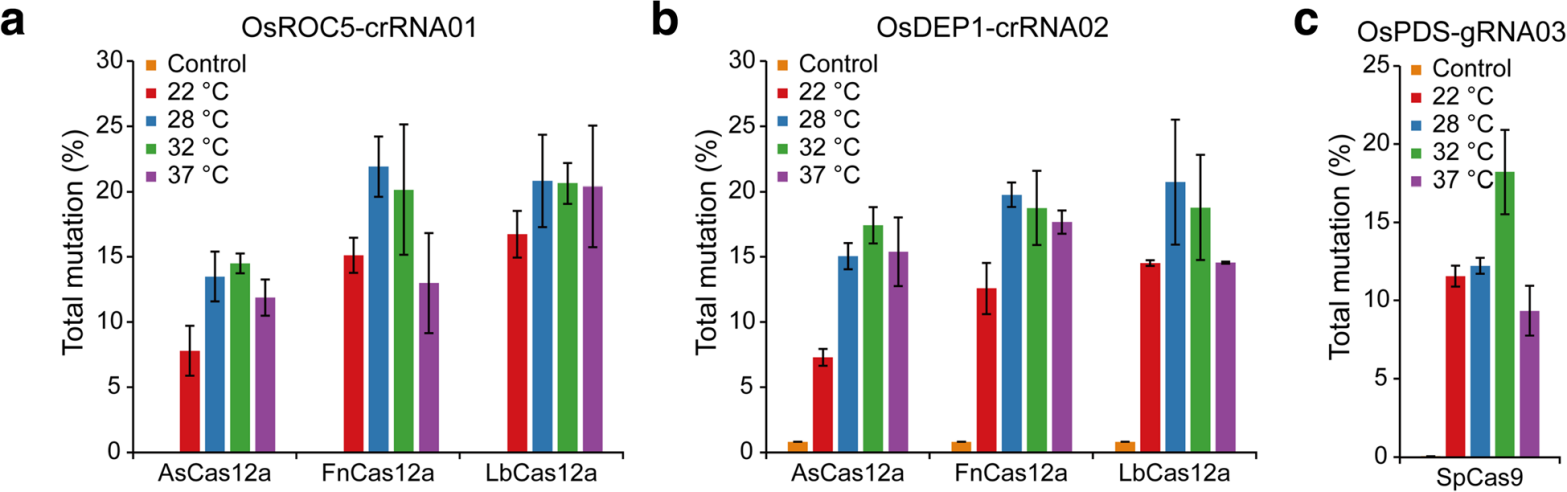
Cas12a and Cas9 nuclease activities in rice protoplasts under different temperatures: 22 °C, 28 °C, 32 °C, and 37 °C. Percentage of mutations by AsCas12a, FnCas12a, and LbCas12a at *OsROC5* (**a**) and *OsDEP1* (**b**), and by SpCas9 at *OsPDS* (**c**). GFP-transfected samples were used as controls. Error bars represent standard deviations of two biological replicates

To investigate temperature sensitivity of AsCas12a, FnCas12a, and LbCas12a, we targeted *OsROC5* and *OsDEP1* in rice protoplasts at 22 °C, 28 °C, 32 °C, and 37 °C. In our system, both Cas12a and the crRNA were expressed under the maize ubiquitin promoter (pZmUbi) and the crRNA was precisely processed by hammerhead (HH) and hepatitis delta virus (HDV) ribozymes [17, 18]. Two biological replicates were first assessed by restriction fragment length polymorphism (RFLP) analysis (Additional file 1: Figure S1) before being submitted for deep sequencing (Fig. 1). For both targets, FnCas12a and LbCas12a were less sensitive to temperatures than AsCas12a. At 22 °C, the mutation frequencies for AsCas12a at both target sites were about 7%, much lower than the rates of FnCas12a and LbCas12a (Fig. 1a, b). The mutation frequencies by AsCas12a, however, were doubled when the transfected protoplasts were incubated at 28 °C, 32 °C, or 37 °C. The improvement on editing efficiency at higher temperatures was also observed for FnCas12a and LbCas12a, but less prominent when compared to AsCas12a (Fig. 1a, b). As a control, we also tested a SpCas9 construct targeting *OsPDS* (Fig. 1c and Additional file 1: Figure S2). Mutation frequencies by SpCas9 at the target site were the same at 22 °C and 28 °C, and the frequency increased at 32 °C (Fig. 1c). Meanwhile, the rice protoplast transfection efficiencies using a DNA vector carrying a green fluorescent protein (GFP) gene were comparable across four temperatures (Additional file 1: Figure S3). The mutation frequencies in SpCas9 and some Cas12a samples decreased at 37 °C (Fig. 1), which was likely caused by heat-related cell abnormalities at this high temperature. The results suggest Cas12a nucleases, like SpCas9, are temperature sensitive in rice cells. A temperature of 28 °C or higher is required for optimal Cas12a activity, while a higher temperature (e.g., 32 °C) is required for optimal Cas9 activity. Notably, AsCas12a is the most temperature sensitive among the three Cas12a nucleases tested.

### Mutation profile analysis of three Cas12a nucleases under different temperatures

**Fig. 2:**
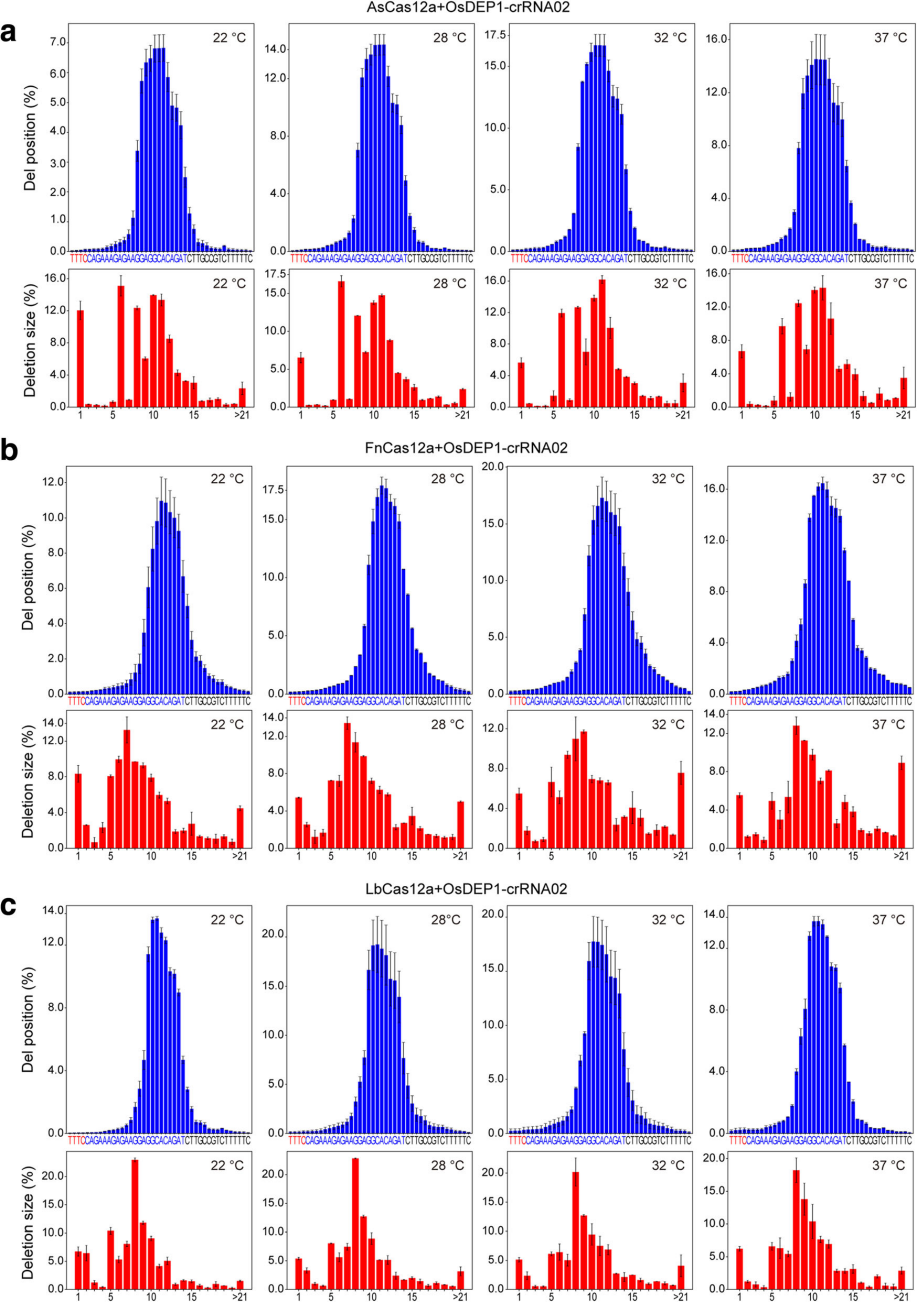
Three Cas12a nucleases generate slightly distinct mutation profiles that are unaffected by temperatures. *OsDEP1* gene targeted by AsCas12a (**a**), FnCas12a (**b**), and LbCas12a (**c**). PAM is red, crRNA is blue. Error bars represent standard deviations of two biological replicates

As targeted mutations are combinational outcomes of the nuclease activity and NHEJ repair, it is important to determine whether NHEJ repair was affected by temperature. We decided to investigate and compare the NHEJ mutation profiles by Cas12a nucleases at different temperatures. Sequence analysis of targeted mutations at *OsDEP1* and *OsROC5* showed differences in deletion size and position among the three Cas12a nucleases (Fig. 2 and Additional file 1: Figure S4), suggesting the staggered DNA ends generated by AsCas12a, FnCas12a, and LbCas12a are slightly different from each other. For example, the predominant deletion sizes (> 10% among all deletions) were 6 bp, 8 bp, 10 bp, and 11 bp for AsCas12a; 7 bp, 8 bp, and 9 bp for FnCas12a; and 8 bp, 9 bp, and 10 bp for LbCas12a. The mutation profiles at the same target site with the same Cas12a nuclease, however, were strikingly similar across four temperatures (Fig. 2 and Additional file 1: Figure S4). Hence, this experiment revealed a different cleavage property of each of the three Cas12a nucleases. However, temperature did not influence NHEJ repair pathway choice (e.g., the use of canonical NHEJ pathway versus the microhomology-based alternative NHEJ pathway), which was further confirmed when we analyzed the NHEJ mutation profiles by Cas9 at different temperatures (Additional file 1: Figure S5).

### High-frequency genome editing by AsCas12a in rice T0 lines under elevated temperature

**Fig. 3:**
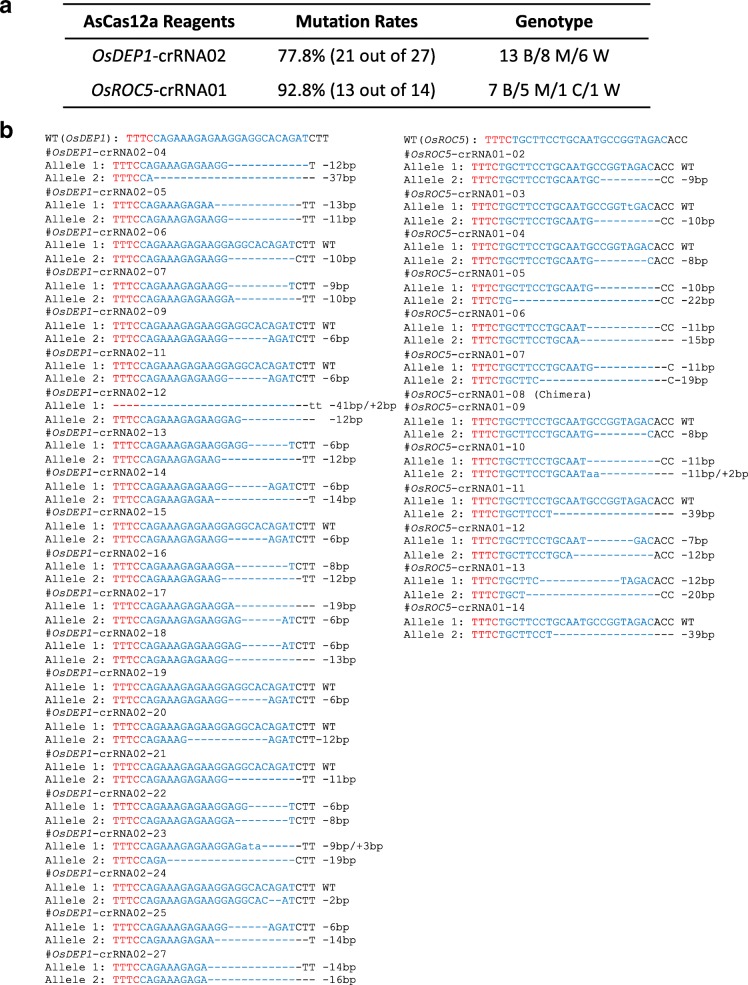
High-temperature treatment resulted in high-frequency genome mutations by AsCas12a in stably transformed rice. **a** Table of mutation rates and observed genotypes at *OsDEP1* and *OsROC5*. B, biallelic; M, monoallelic; W, wild type. **b** Sequences of individual mutated T1 rice plants at the target site. PAM is in red and crRNA in blue

Despite previous attempts [15, 17, 19], AsCas12a has not been successfully applied for generating heritable mutations in plants. To determine whether AsCas12a can be effectively used for genome editing under high-temperature treatments, stable transgenic T0 rice lines targeting *OsDEP1* and *OsROC5* were generated by co-culturing with *Agrobacterium* at 25 °C, selecting at 32 °C, and regenerating shoots at 28 °C. The T0 plants were analyzed by RFLP-based genotyping (Additional file 1: Figure S6) and Sanger sequencing (Fig. 3). The editing frequencies in T0 lines at *OsDEP1* and *OsROC5* were 77.8% and 92.8%, respectively (Fig. 3a and Additional file 1: Figure S6). For *OsDEP1*, 61.9% mutants were biallelic, and for *OsROC5*, 46.2% mutants were biallelic. Nearly 100% mutations in these T0 lines were deletions (Fig. 3b), which was consistent with the deletion size analysis in protoplasts (Fig. 2 and Additional file 1: Figure S4). The data suggest that when a high-temperature regime is applied during rice tissue culture, AsCas12a can reliably generate targeted mutations at high frequencies.

### LbCas12a is very sensitive to temperature in *Arabidopsis*

We next sought to generate heritable mutations in the dicot model plant, *Arabidopsis*, in which Cas12a-based mutagenesis has not been demonstrated. We decided to use a dual Pol II promoter for the LbCas12a system because it resulted in high mutation frequencies in rice [17] and its activity seems less sensitive to temperature (Fig. 1). The pZmUbi promoter used to express LbCas12a, and the crRNA here was also previously applied for Cas9 genome editing in *Arabidopsis* with high efficiency [31]. We targeted *GL2* (*GLABRA 2*) and *TT4* (*TRANSPARENT TESTA 4*) genes with one crRNA for each gene. The WT *Arabidopsis* plants (Col) were transformed with two corresponding T-DNA constructs via the floral dip method. The *Arabidopsis* plants were grown at 22 °C, a temperature at which LbCas12a displayed a good editing activity in rice (Fig. 1a, b). T1 transgenic plants were screened by RFLP, as mutations induced by LbCas12 would likely abolish the restriction enzyme site of *Sal*I at both *GL2* and *TT4* target sites. However, we could not identify a single LbCas12a T1 plant that seemed to carry targeted mutations at either site (data not shown).

**Fig. 4:**
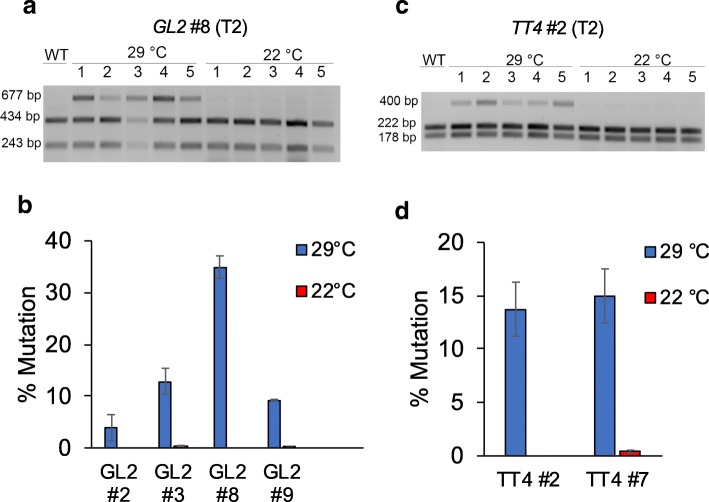
Activity of LbCas12a is highly sensitive to temperature in *Arabidopsis* somatic cells. Examples of RFLP gel of GL2 line #8 (**a**) and TT4 line #2 (**c**). Presence of a third band indicates mutations. Mutation percentages of GL2 lines #2, #3, #8, and #9 (**b**) and TT4 lines #2 and #7 (**d**; plants grown at 29 °C (blue) or 22 °C (red)). Error bars represent standard deviations of five biological replicates

The surprising results prompted us to investigate whether LbCas12a requires a higher temperature to be active in *Arabidopsis*. To test this hypothesis, we decided to try 29 °C as a high temperature because Cas12a nucleases required a temperature of 28 °C or higher to reach optimal activity in rice (Fig. 1). We harvested seeds of T2 population from selected T1 lines. These T2 lines were grown on MS-hygromycin selection medium at 22 °C and 29 °C for 2 weeks, and then were used for DNA extraction followed by RFLP-based mutation analysis. For *GL2*, T2 line #8 showed mutations at 29 °C, but not at 22 °C (Fig. 4c). Further quantified data indicated a mutation frequency of 35% at 29 °C for T2 line #8 (Fig. 4b). Analysis of three additional *GL2* T2 lines all revealed detectable mutation frequencies at 29 °C, but not at 22 °C (Fig. 4b). Similarly, while two examined T2 lines (#2 and #7) for *TT4* showed mutation frequencies of about 14% and 15% at 29 °C, no mutation could be detected at 22 °C (Fig. 4c, d). We also tested a late treatment of transgenic plants at 29 °C (see the “Methods” section), which was less effective in inducing LbCas12a-mediated mutations at both target sites (Additional file 1: Figure S7). In addition, we compared LbCas12a and AsCas12a at editing the *TT4* locus in protoplasts made from *Arabidopsis* rosette leaves. LbCas12a showed higher editing activity at 29 °C than at 22 °C (Additional file 1: Figure S8a). However, editing activity of AsCas12a was undetectable at this locus under both temperatures (Additional file 1: Figure S8b).

### Germline editing by LbCas12a in *Arabidopsis* with high-temperature treatment

**Fig. 5:**
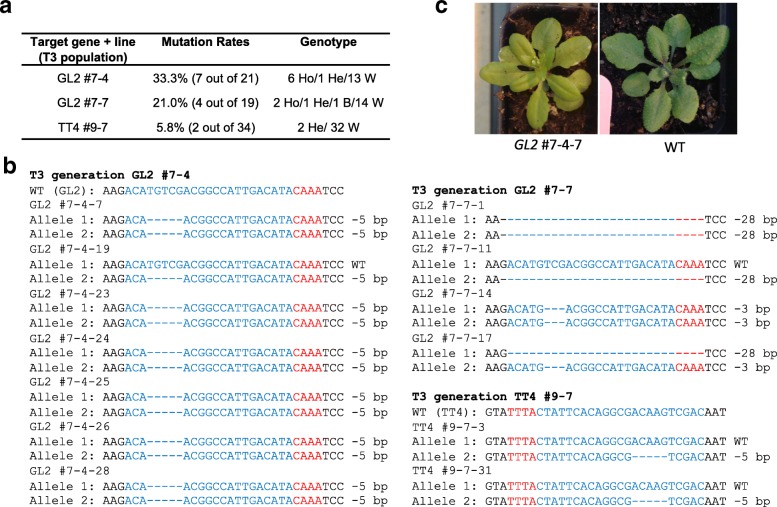
Germline mutagenesis by LbCas12a in *Arabidopsis* with high-temperature treatment. **a** A summary of genotyping results from GL2 #7-4, GL2 #7-7, and TT4 #9-7. Ho, homozygous; He, heterozygous; B, biallelic; W, wild type. **b** Target site sequences of *gl2* mutants from parental line #7-4, #7-7, and sequences of *tt4* mutants. **c** Images of *gl2* mutant (GL2 #7-4-7) and wild type *Arabidopsis* (WT)

We then developed a high-temperature treatment regime for *GL2* and *TT4* T2 plants with early treatment of 29 °C for about 4 weeks before moving the plants to 22 °C for recovery. T3 plants from these T2 parents were grown in a greenhouse at ~ 22 °C to demonstrate stable germline editing and evaluate mutation frequencies. Leaf tissues were collected from the three lines (GL2 #7-4, GL2 #7-7, and TT4 #9-7) for RFLP and Sanger sequencing (Fig. 5a, b). Thirty-three percent of the plants from GL2 #7-4, 21% of plants from GL2 #7-7, and 5.8% from TT4 #9-7 had targeted mutations (Fig. 5a). Among T3 lines from GL2 #7-4, the same mutation (a deletion of 5 bp) was found in all the homozygous and heterozygous plants, suggesting germline transmission from the parent (Fig. 5b). All homozygous GL2 #7-4 plants had a trichome-less (*gl2* loss-of-function) phenotype (Fig. 5c). For GL2 #7-7 T3 plants, only GL2 #7-7-1 showed a loss-of-function phenotype. Sequencing analysis showed that the other homozygous or biallelic plants (e.g., GL2 #7-7-14 and GL2 #7-7-17) had the same allele of 3-bp deletion, suggesting the mutated protein, albeit missing one amino acid, is still functional. The LbCas12a reagent seems less efficient at the *TT4* locus since only two heterozygous mutants were identified out of 34 TT4 #9-7 T3 lines (Fig. 5a, b). We followed two homozygous GL2 #7-4 lines to the T4 generation and found the mutations were germline transmitted (data not shown). Together, we demonstrated LbCas12a-mediated germline editing in *Arabidopsis* with a high-temperature treatment.

### High-frequency genome editing by LbCas12a in maize at 28 °C

**Fig. 6:**
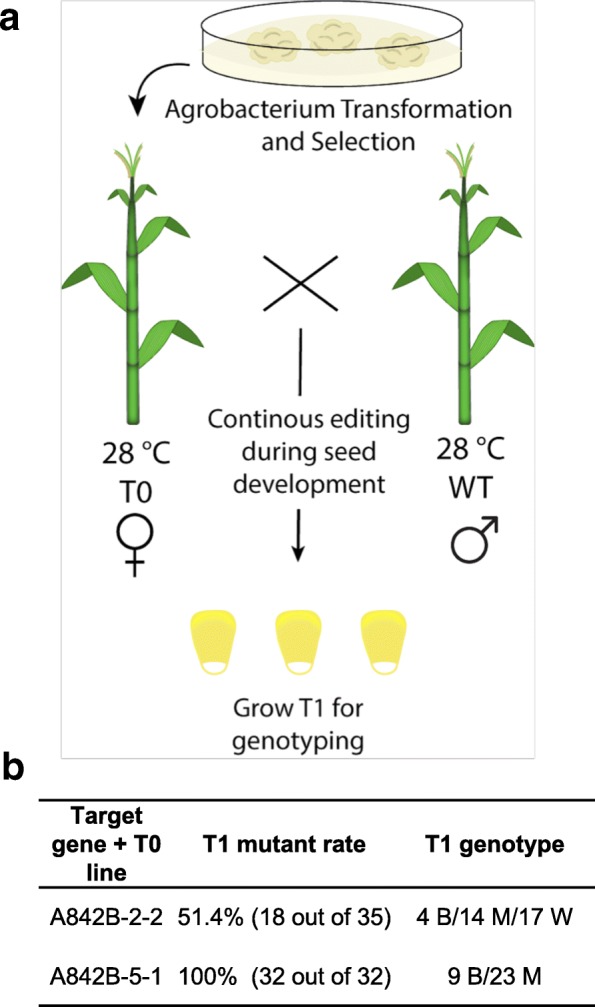
High-temperature regime in maize T0 transgenic plants enables high-frequency mutagenesis by LbCas12a. **a** Diagram of maize transformation and heat treatment. Transgenic plants are crossed with a wild type inbred B104 pollen donor. B, biallelic; M, monoallelic; W, wild type. **b** Mutation rates and genotyping results of T1 generation from two maize mutant lines, A842B-2-2 and A842B-5-1

Our data in rice and *Arabidopsis* collectively suggest a temperature around 28 °C could be an optimal and practical temperature for achieving high-efficiency plant genome editing by Cas12a nucleases. To validate whether this is widely applicable to other plant species, we further tested high-temperature regimes in maize, an important commodity crop in which we recently demonstrated genome editing with LbCas12a [20]. We grew two independent T0 lines expressing LbCas12a and ZmGL2-crRNA1 (A842B-2-2 and A842B-5-1), along with the WT plants, in a greenhouse with a 16-h/8-h photoperiod and the temperature setting of 28 °C/21 °C (day/night). These T0 plants were crossed with WT B104 lines (the pollen donor) and kept in the same environment until maturity for harvesting T1 seeds (Fig. 6a). We genotyped T1 seedlings by Sanger sequencing (Fig. 6b). For A842B-2-2, 51.4% of T1 plants were mutated (18 out of 35). For A842B-5-1, 100% of T1 plants were mutated (32 out of 32), suggesting high mutagenesis frequencies. Biallelic mutants were obtained in T1 plants, and many new mutations were generated in the T1 generation (Fig. 6b and Additional file 1: Figure S9), indicating high activity of LbCas12a in maize at 28 °C.

### Probing Cas12a temperature sensitivity with a dLbCas12a-SRDX repressor

**Fig. 7:**
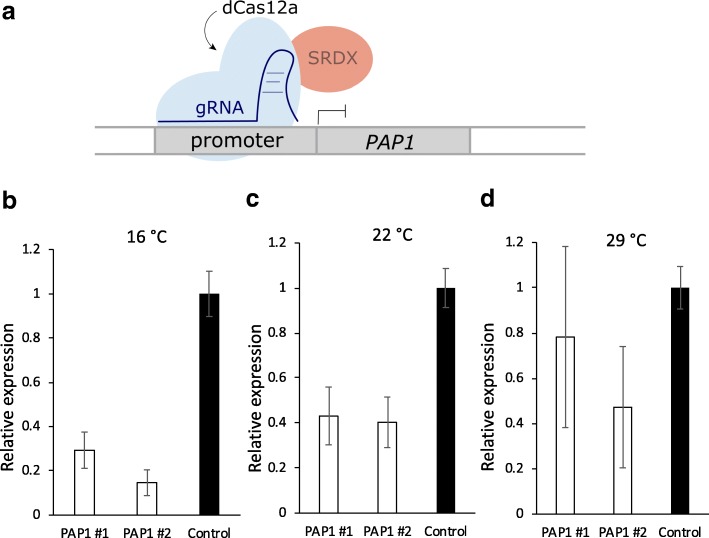
Quantitative real-time (qRT)-PCR showing dLbCas12a-mediated transcriptional repression in *Arabidopsis* at different temperatures. **a** Diagram of a dCas12a-SRDX repressor targeting *PAP1*. Relative expression of *PAP1* mRNA, normalized to *EF1α*, for two lines at 16 °C (**b**), 22 °C (**c**), and 29 °C (**d**). Error bars represent standard errors of four biological replicates from dCas12a-SRDX transgenic lines PAP #1 and #2, and three biological replicates from WT control plants

While the above-mentioned results clearly demonstrated activities of Cas12a nucleases are temperature sensitive, it is less clear which of the underlining mechanisms are affected. Before Cas12a can cleave DNA, it must scan the genome, find a PAM and crRNA complimentary sequence, unwind the DNA, and make conformational changes that activate nuclease activity [32, 33]. Temperature may affect any one, or all, of these steps. Although testing all these in vivo will be challenging, we reasoned that we could test whether Cas12a *in planta* binding to DNA is affected by temperature. Given the contrasting differences of LbCas12a editing activities in *Arabidopsis* at 22 °C and 29 °C, we decided to test this in *Arabidopsis* by using a dLbCas12a-SRDX repressor that we had previously developed [17]. We constructed a dLbCas12a-SRDX vector to target the promoter of *PAP1* (*PRODUCTION OF ANTHOCYANIN PIGMENT 1*) with a single crRNA (Fig. 7a). Successful transcriptional repression would indicate successful binding of deactivated LbCas12a (dLbCas12a) to the target DNA. We selected two independent T1 lines for obtaining T2 generation seeds. T2 plants were germinated in MS-hygromycin selection medium for a week and then transplanted to new MS medium for 1-week treatment at 16 °C, 22 °C, and 29 °C, before analysis for gene expression by quantitative real-time (qRT)-PCR. The highest levels of repression were seen at 16 °C, where the expression of *PAP1* was reduced to 30% and 20% of WT expression in the two transgenic lines, respectively (Fig. 7b). At 22 °C, expression of *PAP1* was reduced to 40% of the WT in both lines (Fig. 7c). At 29 °C, *PAP1* was also repressed, but more variation was seen between the two lines (Fig. 7d). Unsurprisingly, a comparative analysis of *PAP1* in control plants shows that expression was higher at 29 °C, likely as a response to heat stress (Additional file 1: Figure S10). This could explain some variation between the lines and larger error bars for the repression results at 29 °C. Because repression was accomplished at lower temperatures, these results suggest that unlike genome editing, binding of LbCas12a to the target DNA is not abolished at lower temperatures. Further analysis of dLbCas12a-SRDX expression in transgenic lines indicated that its mRNA level was not elevated at a higher temperature (e.g., 29 °C) (Additional file 1: Figure S11). Rather, PAP1 #2 line showed higher expression of dLb-cas12a-SRDX at 16 °C (Additional file 1: Figure S11), which is consistent with stronger transcriptional repression on the target gene (*PAP1*) observed in this line at 16 °C (Fig. 7b).

## Discussion

In this study, we investigated temperature sensitivity of different Cas12a nucleases and applied high-temperature treatment for genome editing in plants. First, we found in rice cells AsCas12a is more sensitive to temperature than LbCas12a, and our results are consistent with the observation in zebrafish [29]. By doing stable rice transformation at a higher temperature, we showed that AsCas12a can induce heritable mutations at frequencies of 77.8% and 92.8% at two target sites. Hence, we not only demonstrated the use of AsCas12a for generating mutated plants for the first time, but also presented an efficient AsCas12a rice genome editing system, with mutagenesis frequencies close to those that we previously established for LbCas12a [17] and FnCas12a [18]. Interestingly, although we found mutation profiles for the three Cas12a nucleases were different from each other in rice cells, the mutation profiles for AsCas12a were much more different from those of FnCas12a and LbCas12a. While an in vitro DNA cleavage assay revealed AsCas12a, LbCas12a, and FnCas12a, all cleave approximately after the 18th base (relative to PAM) on the non-targeted strand and after the 23rd base on the targeted strand [3], genome-wide studies in human cells also suggested different mutation repair profiles of AsCas12a and LbCas12a [34, 35]. Further, both in vitro and in vivo data have suggested that AsCas12a has higher targeting specificity than LbCas12a [3, 34]. More recently, kinetic basis for high targeting specificity of AsCas12a was revealed [36]. These previous studies highlighted the importance of developing AsCas12a-based genome editing systems, supporting the significance of our work on improving AsCas12a activity in plants. We hope our success with AsCas12a in rice will facilitate future research into applying and improving AsCas12a for genome editing in other plant species.

We also observed temperature sensitivities for LbCas12a in *Arabidopsis*. While LbCas12a showed reasonable nuclease activities in rice protoplasts and detectable activity in *Arabidopsis* protoplasts at 22 °C, it barely worked in *Arabidopsis* cells of stable transgenic lines at the same temperature. This could be due to the procedures of delivering CRISPR-Cas12a reagents, in vitro cell culture for the transient protoplast assay and floral dip for *Arabidopsis*. Also, the length of Cas12a treatment and the tissue sources for evaluating mutagenesis are different among transient and stable transgenesis. It is possible that any of these factors had contributed to the drastic difference between rice and *Arabidopsis* on editing efficiencies we observed. It is likely that chromatin structure plays a role as it has been shown to impact genome editing. For example, the natural cycle of nucleosome breathing and ATP-driven chromatin remodelers are essential for Cas9 binding at target sites [37, 38], and the same can be true for Cas12a. Recent comparative studies have revealed distinct chromatin packing in rice and *Arabidopsis* [39, 40]. It will be interesting to investigate how chromatin states in different plant species impact Cas12a and other CRISPR-Cas systems on genome editing. Nevertheless, we were able to rescue LbCas12a activity at a higher temperature and demonstrated LbCas12a-based germline editing in *Arabidopsis*. By contrast, AsCas12a showed undetectable editing activity at the *TT4* locus in *Arabidopsis* protoplasts, which is consistent with the general observation in rice where AsCas12a has lower activity than LbCas12a. Since the baseline genome editing efficiency in *Arabidopsis* is much lower than that in rice, more future improvement is required in order to establish an efficient genome editing system in *Arabidopsis* using AsCas12a. Since we did not test the effects of temperature change on AsCas12a in rice seedlings, it is possible that using high temperature at different stages (e.g., seedlings vs calli) may result in different effects.

We also applied a high-temperature regime for achieving LbCas12a-based high-frequency genome editing in maize T1 lines. We showed that when transgenic maize lines expressing LbCas12a and crRNA were crossed to the WT plants, mutated T1 lines can be generated at a frequency as high as 100%, provided that the entire process of generating the T1 generation is done at a day time temperature of 28 °C. Many of the mutations discovered in the T1 lines are de novo generated new mutations. Our data were consistent with the recent report that new mutations could be generated in gametes or zygotes by Cas9 in maize [41]. Although we crossed our LbCas12a lines to WT, it is conceivable that we can also cross these transgenic lines to other transformation-recalcitrant maize varieties to knock out genes in different maize genetic backgrounds, as was shown previously with Cas9 [42]. Following our recent demonstration of LbCas12a in maize [20], the work here further streamlined an efficient LbCas12a system for targeted mutagenesis in maize.

In addition, we showed that Cas9 is temperature sensitive in rice, which is consistent with the recent report that investigated this issue in *Arabidopsis* and citrus [30]. Interestingly, Cas9 showed similar nuclease activities at 22 °C and 28 °C, and we started to see increased nuclease activity in rice protoplasts at 32 °C (Fig. 1c). Cas9 displayed optimal nuclease activity in human cells at 37 –39 °C [43], and a heat stress of 37 °C was applied for improving Cas9 editing in plants [30]. In zebrafish, AsCas12a had poor activity at 28 °C and its activity was drastically improved by elevating the temperature to 34 °C [29]. By contrast, we found Cas12a nucleases seem to reach optimal activities in plants at around 28–29 °C, which is more feasible given most plants grow in temperatures around 22–29 °C. For example, we have grown rice and maize constantly at 28 °C and have treated *Arabidopsis* at 29 °C for up to ~ 4 weeks continuously. However, lengthy and continuous treatment at 29 °C significantly impedes *Arabidopsis* growth. Further exploration of heat treatment regimens will probably result in more robust genome editing in plants. Hence, while Cas12a nucleases are temperature sensitive, it should not be a barrier that prevents adoption of them for genome editing in many other plant species.

A question remains, however, as why Cas12a-based genome editing is temperature sensitive. With the analysis of NHEJ mutations by all three Cas12a nucleases across four different temperatures, we ruled out the possible involvement of DNA repair pathways in this difference. Opposite effects of high temperatures on the activities of ZFN and TALEN versus CRISPR-Cas9 were reported in mammalian cells [43], which also suggested the effects were unlikely due to DNA repair machinery. Using a dLbCas12a-SRDX repressor, we further demonstrated that the DNA binding property of LbCas12a at lower temperatures is as good as, if not better than, higher temperatures. This is consistent with our previous observation that the dAsCas12a-SRDX repressor could work robustly in mediating targeted transcriptional repression at room temperature in *Arabidopsis* [17]. While DNA binding is not significantly affected under these temperatures, it is still possible that chromatin structure is affected by temperature in a way that impacts the necessary conformation change of the Cas12a/crRNA ribonucleoprotein complex that is required for activation of nuclease activity, as supported by the data in zebrafish [29]. Our data collectively points to a working hypothesis that Cas12a nuclease activity is affected by temperature. Upon activation, Cas12a proteins also unleash single-stranded DNase activities [44, 45]. We predict such non-specific DNase activities are likely also temperature sensitive. Finally, given that we have narrowed down the main cause of temperature sensitivity to Cas12 nuclease activities, it will be highly valuable and should also be possible to engineer Cas12a variants that are more active at lower temperatures, similar to engineering Cas12a variants with altered PAM specificities [25], which we and others have recently demonstrated in plants [18, 26].

## Methods

### T-DNA vector construction

T-DNA vectors for CRISPR-Cas9 were constructed based on the protocols described previously [46]. Briefly, forward and reverse oligos for OsPDS-gRNA03 (Additional file 1: Table S1) were phosphorylated, annealed, and ligated into pTX172 (Addgene #89259) at its *Bsa*I sites.

T-DNA vectors for CRISPR-Cas12a were constructed based on the protocols described previously [17]. Forward and reverse oligos for OsROC5-crRNA01, OsDEP1-crRNA02, AtGL2-crRNA1, and AtTT4-crRNA1 (Additional file 1: Table S1) were phosphorylated, annealed, and cloned into the *Esp3I* sites of pYPQ141-ZmUbi-RZ-As (Addgene #86196), pYPQ141-ZmUbi-RZ-Lb (Addgene #86197), and pYPQ141-ZmUbi-RZ-Fn (Addgene #108864). The resulting crRNA expression vectors were mixed with pYPQ203 (destination vector, Addgene #86207) and with pYPQ220 (AsCas12a, Addgene #86208), pYPQ230 (LbCas12a, Addgene #86210), and pYPQ239 (FnCas12a, Addgene #108859), to generate the final T-DNA binary vectors using multi-site LR reactions (1-5-2) [47, 48]. The LbCas12a-ZmGL2 T-DNA vector (A842B) for maize transformation was described previously [20].

The T-DNA vector for transcriptional repression in *Arabidopsis* was constructed similarly. The protospacer of AtPAP1-crRNA1 (Additional file 1: Table S1) was cloned into pYPQ141-ZmUbi-RZ-Lb at *Esp3I* sites in the form of phosphorylated and annealed oligos. Then, a multi-site LR reaction using pYPQ141-ZmUbi-RZ-Lb-AtPAP1-crRNA1, pYPQ233 (dLbCas12a-SRDX, Addgene #86211), and pYPQ202 (Addgene #86198) was conducted to generate the final T-DNA vector.

### Rice protoplast transfection and stable transformation

The Japonica rice cultivar Nipponbare was used in this study. Polyethylene glycol (PEG)-mediated transfection of rice mesophyll protoplasts with T-DNA vectors was carried out according to our previously published protocol [46, 49]. After transfection, rice protoplasts were divided and incubated for 2 days at four temperatures, 22 °C, 28 °C, 32 °C, and 37 °C. A DNA construct carrying GFP marker gene was used as a control to determine the transfection efficiencies. Cells with green fluorescence were counted 2 days after the transfection.

Rice stable transformation was conducted as published previously [46]. Plants were grown in a growth chamber at 25 °C for co-culture, 32 °C for selection, and 28 °C for shoot generation.

### Mutagenesis analysis at target sites

Genomic DNA was extracted from transfected rice protoplasts or leaves of transgenic lines using the cetyl trimethylammonium bromide (CTAB) method [50]. The genomic region flanking target sites were PCR amplified with primers DEP1-F 5′-TCACCGATTCTTTCCATGCG-3′, DEP1-R 5′-GCCACAATCGGGTTTGCATT-3′, ROC5-F 5′ CTTATGTTCCGTTCCAATCCT-3′, ROC5-R 5′ CCTACACTTCACATTTCCACCT-3′ and PDS-F 5′ GCTCACACTGTTTTGTCGTCC-3′, and PDS-R 5′ ATCATATGCAGCGCTGGAGT-3′. *DEP1* PCR products were digested with *Bgl*II and *ROC5* PCR products were digested with *Nla*III for RFLP analysis. *PDS* PCR products were digested with *AseI*. The PCR products were digested by their corresponding restriction enzymes overnight and then analyzed by agarose gel electrophoresis. Mutations in T0 plants were further identified by Sanger sequencing of the PCR products.

### High-throughput sequencing analysis

High-throughput sequencing analysis was conducted for transfected rice protoplast samples as published previously [46, 51]. The genomic region flanking target sites were PCR amplified using barcoded primers. Purified DNA samples were quantified by Qubit 2.0 Fluorometer (Life Technologies) and were sequenced using Illumina HiSeq 2500 platform. Data processing was carried out using CRISPRMatch [52].

### *Arabidopsis* stable transformation, temperature treatment, and mutation analysis

*Arabidopsis* WT plants (Col) were transformed by the floral dip method [53]. Seeds for T1, T2, and T3 generations were sterilized using 50% bleach and 0.05% Tween, vernalized at 4 °C for 3 days, then plated to MS-hygromycin plates. After a week, transgenic plants were transferred to MS clean plates for a week of recovery before soil transplantation. To test a variety of T2 lines, five individual plants were sacrificed after 2 weeks of heat treatment at 29 °C on MS plates. Leaf tissue was used for DNA extraction using a modified CTAB method [50]. The rest of the plants were transferred to soil and kept at 29 °C for a total of 29 days before recovery at 22 °C. A second batch of plants was heat treated to test an alternative method of late treatment at 29 °C. For this, 6 days after plating, the plants were kept at 29 °C for 8 days. After 24 days of recovery at 22 °C, plants were kept at 29 °C again for 14 days.

For mutation analysis, a ~ 677 bp fragment covering the *GL2* target site was amplified using the primers GL1-F1 5′-GATGGCTGCCAATGCTGTAGCTGG-3′ and GL2-R1 5′-CGTCAACTACTCTTCTGCCCAGG-3′, and a ~ 400-bp fragment covering the *TT4* target site was amplified using the primers TT4-F2 5′-AGGCATCTTGGCTATTGGCACTG-3′ and TT4-gR3-top 5′-gattGGGCTGGCCCCACTCCTTGA-3′. *GL2* and *TT4* PCR products underwent RFLP analysis through direct digestion with restriction enzyme *Sal*I and analysis on 1.5% and 2% agarose gels, respectively. The mutation percentages for each plant were estimated from RFLP gels with Image Lab (BioRad). PCR products were cleaned using Exonuclease I and Antarctic Phosphatase and sent for Sanger sequencing. Results were aligned in Snapgene and decoded with CRISP-ID (GSL Biotech LLC) [54].

### *Arabidopsis* protoplasts isolation and transformation

*Arabidopsis* protoplast isolation and transformation were based on our previous study [49, 55]. *Arabidopsis* Col-0 plants were grown under 12-h light/12-h dark photoperiod at 22–25 °C for 4 weeks. About 10–20 fresh leaves were cut into leaf strips by razor blades at 0.5–1 mm in width. Leaf strips were quickly transferred into 8–10 ml enzyme solution (1.0–1.5% Cellulase R10, 0.25% Macerozyme R10, 0.4 M Mannitol, 20 mM MES, 20 mM KCl, 10 mM CaCl2, 0.1% BSA, and pH 5.8), followed by vacuum infiltration for 30 min. The leaf strips were digested by shaking at 30 rpm for 1–4 h at 25 °C in the dark. The digested products were filtered with 70-μm nylon mesh into 50-ml tube with 10 ml W5 buffer. The protoplasts were collected at 200×*g* for 2 min. Then, they were resuspended with 10 ml W5 buffer twice and were centrifuge at 100×*g* for 1 min at RT. The cells were suspended in MMG buffer (0.4 M Mannitol, 4 mM MES, 15 mM MgCl_2_, pH 5.8) for 1 × 10^5^ cells/ml. Two hundred microliter protoplasts were mixed with 30 μl plasmid (30 μg) and 230 μl PEG buffer (40% *w*/*v* PEG4000, 0.2 M mannitol, and 0.1 M CaCl_2_) for incubation at 23 °C for 30 min. After adding 900 μl W5 buffer to stop transformation, the protoplasts were centrifugal at 200×*g* for 5 min and resuspended in 1 ml WI buffer (0.5 M mannitol, 20 mM KCl, and 4 mM MES at pH 5.7), and then transferred into two six-well culture plates in equal amount and incubated at 22 °C and 29 °C. After incubation for 24 h, the protoplasts were collected for DNA extraction and further analysis.

### Transgenic T1 maize seeds, temperature treatment, and mutation analysis

Transgenic maize T1 seeds originated from two maternal T0 lines carrying a LbCas12a construct and on-target mutations in the target gene *GL2* (A842B-2-2 and A842B-5-1) [20]. These maternal T0 lines had been crossed with wild-type B104 pollen donors. The T0 lines were grown in a greenhouse with a 16-h/8-h photoperiod, and the temperature setting of 28 °C/21 °C (day/night). For each T0 line, 20 T1 seeds were sown on a tray containing Metro-Mix 900 potting mix (Sun-Gro Horticulture, Agawam, MA). Leaf tissues, first and second leaves, were collected from each T1 plant and pooled for genomic DNA isolation. The presence of T-DNA was confirmed by PCR using the primers LbCas12a-F 5′-AATGGAACGCGGAGTATGAC-3′ and LbCas12a-R 5′-ACATGTCGCCCTTCTTGAAC-3′ [20]. The genotyping of the T1 plants were performed by PCR and sequencing using the primers Zm-gl2-F2 5′-CACAGCCTTGCAATCAATTC-3′, Zm-gl2-R2 5′-GCTGACGTGGAAGGAGTAGC-3′, and ZmGl2-exon2-F1 5′-ACACCGTGTCTTCGTCAAAA-3′ [20]. About 1 kb fragment flanking, the LbCas12a target site was amplified using the oligonucleotides Zm-gl2-F2 and Zm-gl2-R2, and single-band amplification was confirmed by 1% agarose gel electrophoresis. PCR products were cleaned up by ExoSAP-IT kit (ThermoFisher Scientific) according to the manufacturer’s instruction. The oligonucleotide ZmGl2-exon2-F1 was used for Sanger sequencing, and the resulting trace files were analyzed by the Tracking of Indels by Decomposition (TIDE) [56] and DSDecode [57].

### Transcriptional repression in *Arabidopsis*

*Arabidopsis* dLbCas12a-PAP1 T2 plants were grown on MS media with hygromycin at 22 °C for a week. They were then transferred to MS medium without hygromycin, allowed to recover at 22 °C for 2 days, and then grown at 16 °C, 22 °C, and 29 °C for a week. *Arabidopsis* leaf tissue was collected from these T2 seedlings. The qRT-PCR analysis was carried out following previously described protocols [47] with minor modifications. GUS transformed plants, also expressing a hygromycin resistance gene, were used as controls. Total RNA was extracted using TRIzol™ Reagent (Invitrogen) following the manufacturer’s instructions with the exception that samples were extracted twice using chloroform and washed twice by 75% ethanol. RNA was treated with DNase I (New England BioLabs) to remove DNA contamination. Complementary DNA (cDNA) was synthesized using SuperScript III First-Strand Synthesis System (Invitrogen) with Oligo dT. The qRT-PCR was set up using Applied Biosystems SYBR Green Master Mix (Invitrogen) and ran on the CFX96 Touch™ Real-Time PCR Detection System. The following primers were used for the transgenic plant samples: PAP1-F 5′-AGTATGGAGAAGGCAAATGGC-3′ and PAP1-R 5′-CACCTATTCCCTAGAAGCCTATG-3′, LbCas12a-RT-F1 5′-TTCGTTCAACGGATTCACAA-3′, and LbCas12a-RT-R1 5′-GCTTGTCAAAAATTGCGTCA-3′. Elongation factor 1 α (EF1α) was used as the internal control and amplified with the following primers: EF-1α-F 5′-TGAGCACGCTCTTCTTGCTTTCA-3′ and EF-1α-R 5′-GGTGGTGGCATCCATCTTGTTACA-3′. The average of three technical replicates was used for data analysis of each biological replicate. Relative expression to controls was calculated using the comparative threshold cycle method.

## Additional file

Additional file 1: **Figure S1.**RFLP analysis of nuclease activities of three Cas12a (Cpf1) nucleases in rice protoplasts under different temperatures. **Figure S2.** RFLP analysis of SpCas9 activity in rice protoplasts under different temperatures. **Figure S3.** Measurement of rice protoplast transfection efficiencies using GFP construct at different temperatures. **Figure S4.** Three Cas12a nucleases generate slightly distinct mutation profiles at *OsROC5* site which are unaffected by temperature. **Figure S5.** Mutation profiles by SpCas9 are largely unaffected by temperature. **Figure S6.** RFLP analysis of T0 mutant lines by AsCas12a. **Figure S7.** Late treatment of high temperature is ineffective on promoting LbCas12a-induced mutations in *Arabidopsis.* **Figure S8.** Analysis of AsCas12a-mediated mutagenesis at the *TT4* locus in *Arabidopsis* protoplasts. **Figure S9.** Genotyping results of T1 maize lines. **Figure S10.** *PAP1* expression at three different temperatures in the GUS control plants (WT). **Figure S11.** *dCas12a-SRDX* expression at three different temperatures in transgenic lines. **Table S1.** Guide RNA oligos. (PPTX 10187 kb)

Additional file 2: Raw data. This file contains raw data with individual data points or replicates for Figs. 1, 2, 4, 7 and Additional file 1 Figure S3, S4, S5, S8, S10, S11. (XLSX 120 kb)
