# Supplementary material for: Application of CRISPR-Cas12a temperature sensitivity for improved genome editing in rice, maize, and Arabidopsis
Source: BMC Biol. 2019 Jan 31;17:9. doi: 10.1186/s12915-019-0629-5 (PMC6357469; doi:10.1186/s12915-019-0629-5)

## Slide 1
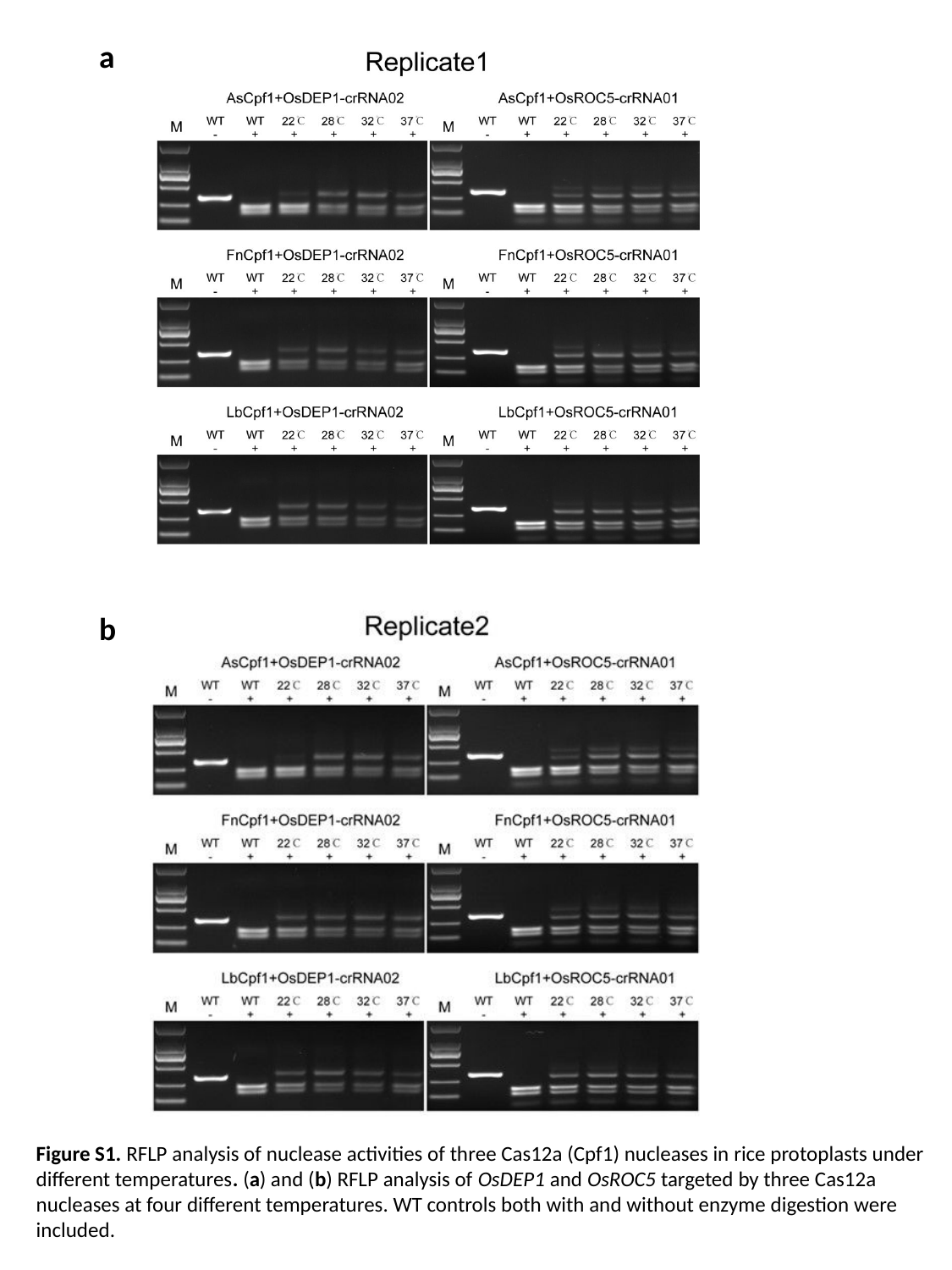

a
b
Figure S1. RFLP analysis of nuclease activities of three Cas12a (Cpf1) nucleases in rice protoplasts under different temperatures. (a) and (b) RFLP analysis of OsDEP1 and OsROC5 targeted by three Cas12a nucleases at four different temperatures. WT controls both with and without enzyme digestion were included.

## Slide 2
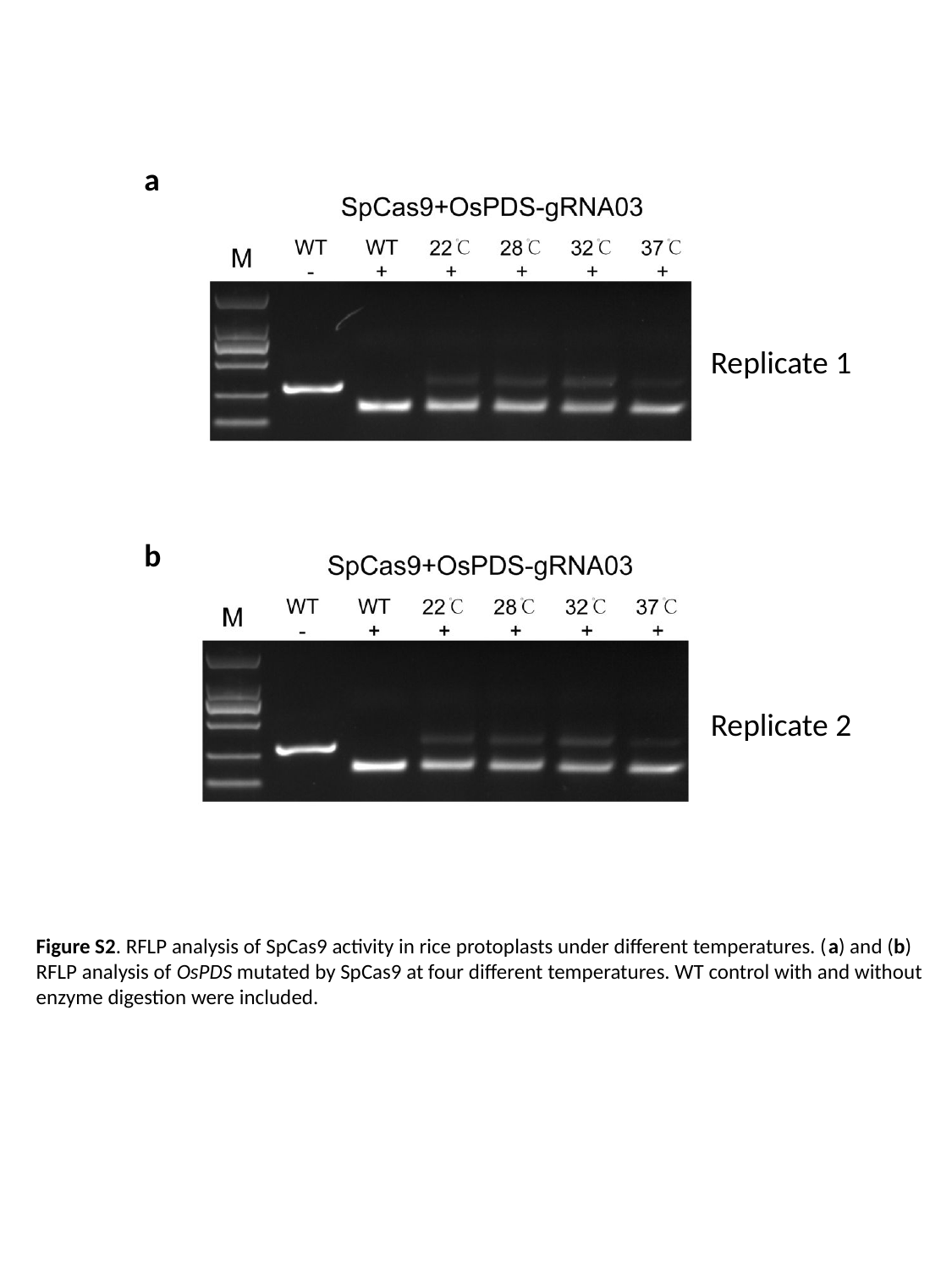

a
Replicate 1
b
Replicate 2
Figure S2. RFLP analysis of SpCas9 activity in rice protoplasts under different temperatures. (a) and (b) RFLP analysis of OsPDS mutated by SpCas9 at four different temperatures. WT control with and without enzyme digestion were included.

## Slide 3
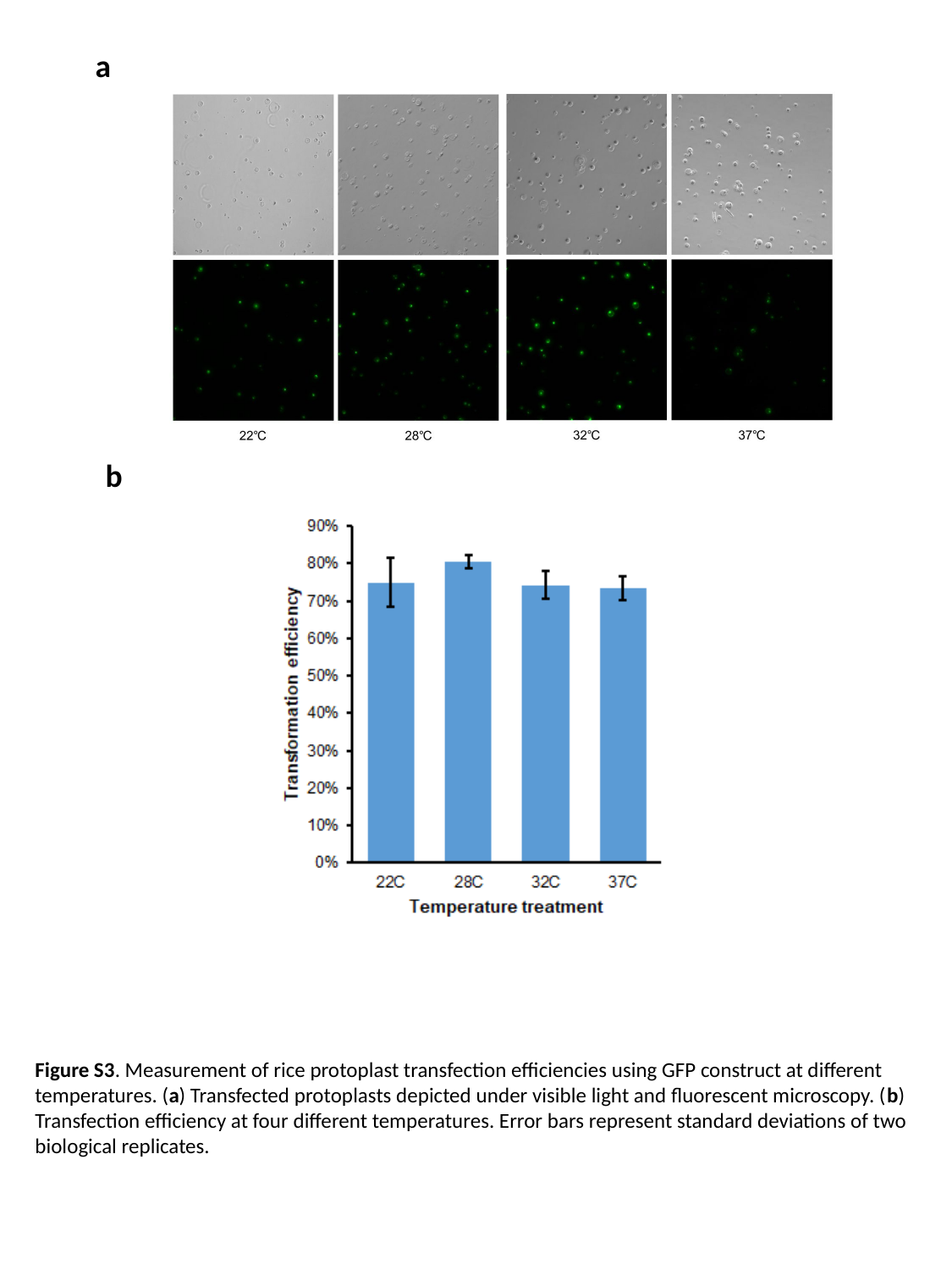

a
b
Figure S3. Measurement of rice protoplast transfection efficiencies using GFP construct at different temperatures. (a) Transfected protoplasts depicted under visible light and fluorescent microscopy. (b) Transfection efficiency at four different temperatures. Error bars represent standard deviations of two biological replicates.

## Slide 4
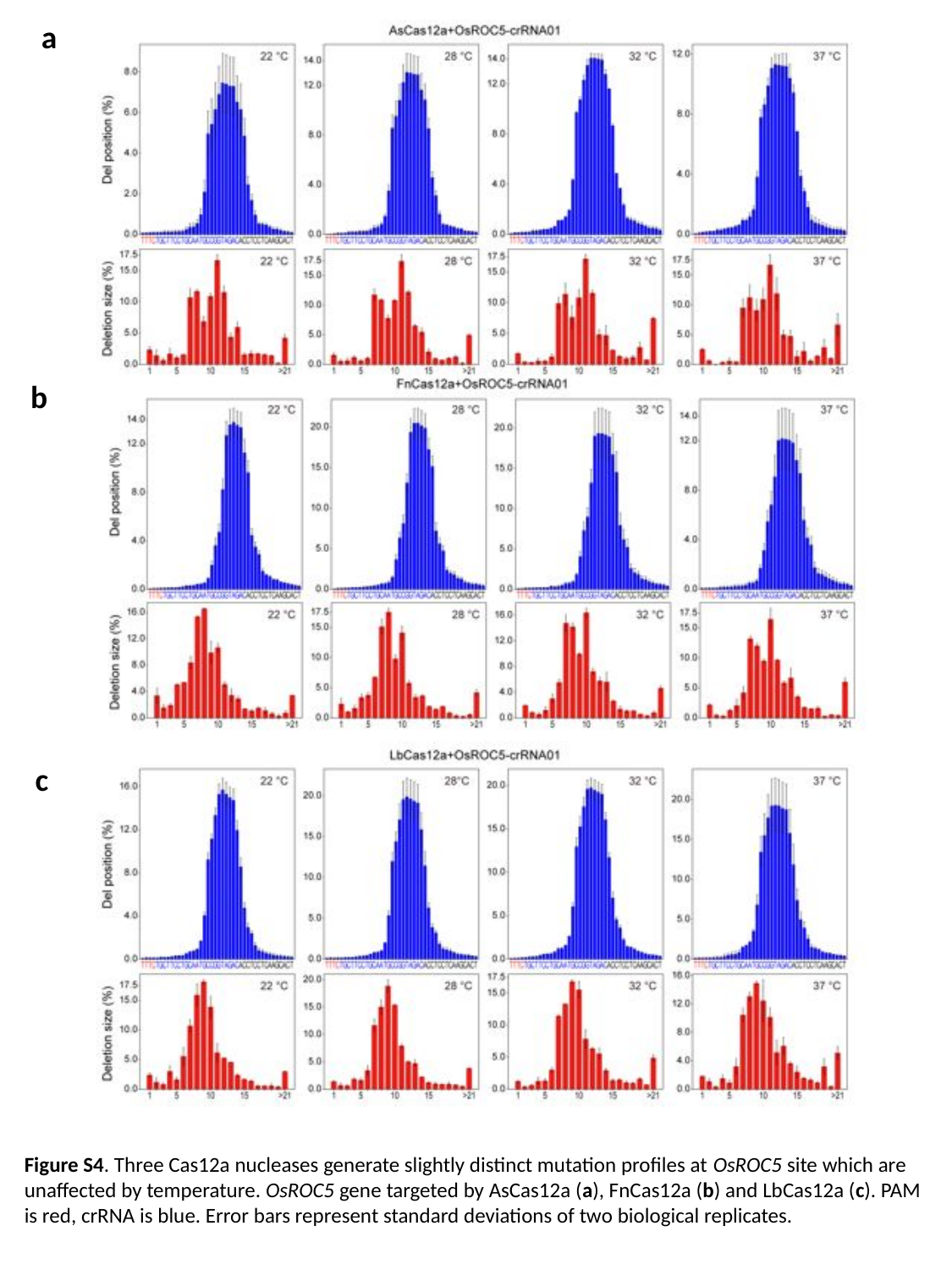

a
b
c
Figure S4. Three Cas12a nucleases generate slightly distinct mutation profiles at OsROC5 site which are unaffected by temperature. OsROC5 gene targeted by AsCas12a (a), FnCas12a (b) and LbCas12a (c). PAM is red, crRNA is blue. Error bars represent standard deviations of two biological replicates.

## Slide 5
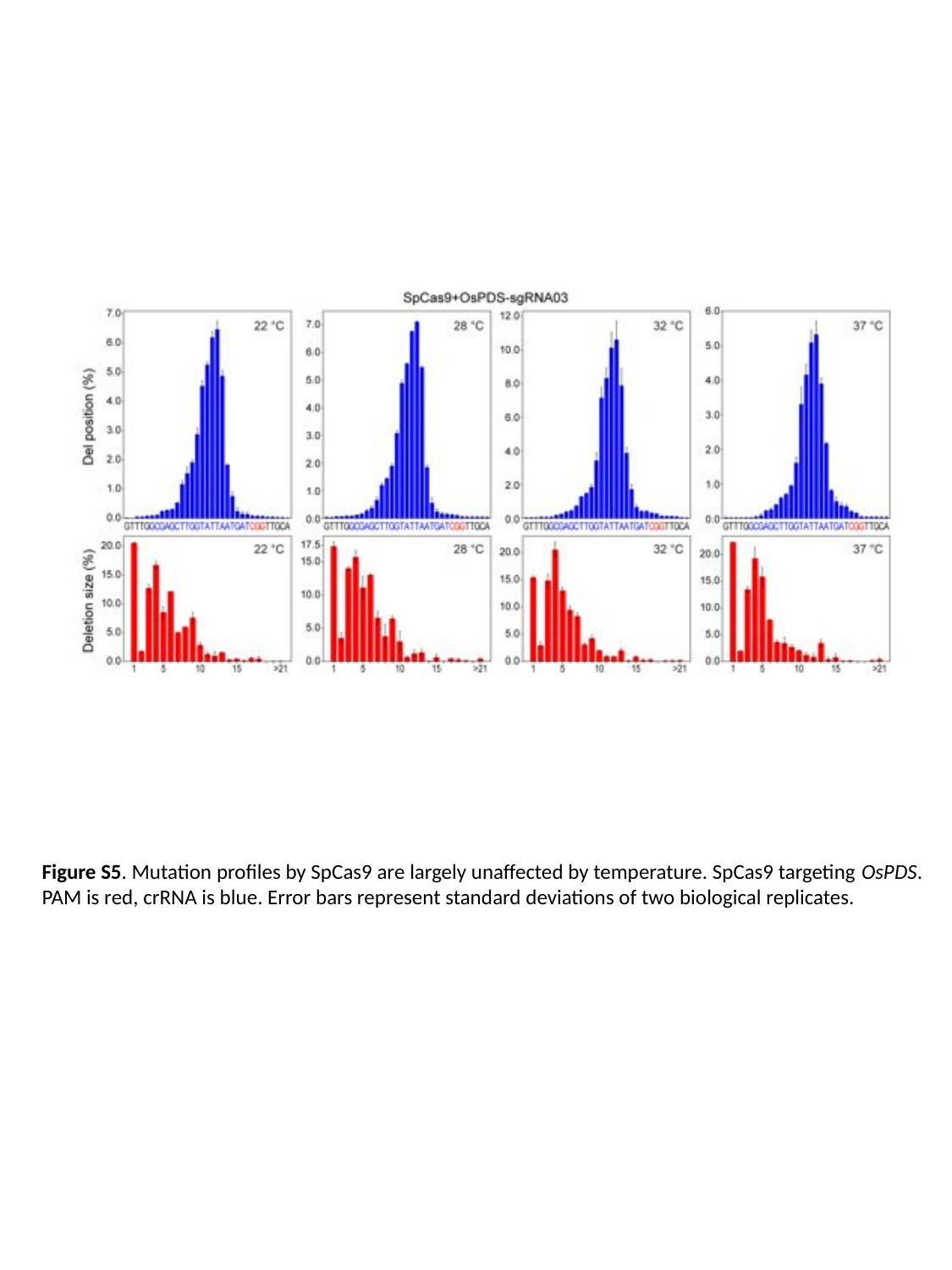

Figure S5. Mutation profiles by SpCas9 are largely unaffected by temperature. SpCas9 targeting OsPDS. PAM is red, crRNA is blue. Error bars represent standard deviations of two biological replicates.

## Slide 6
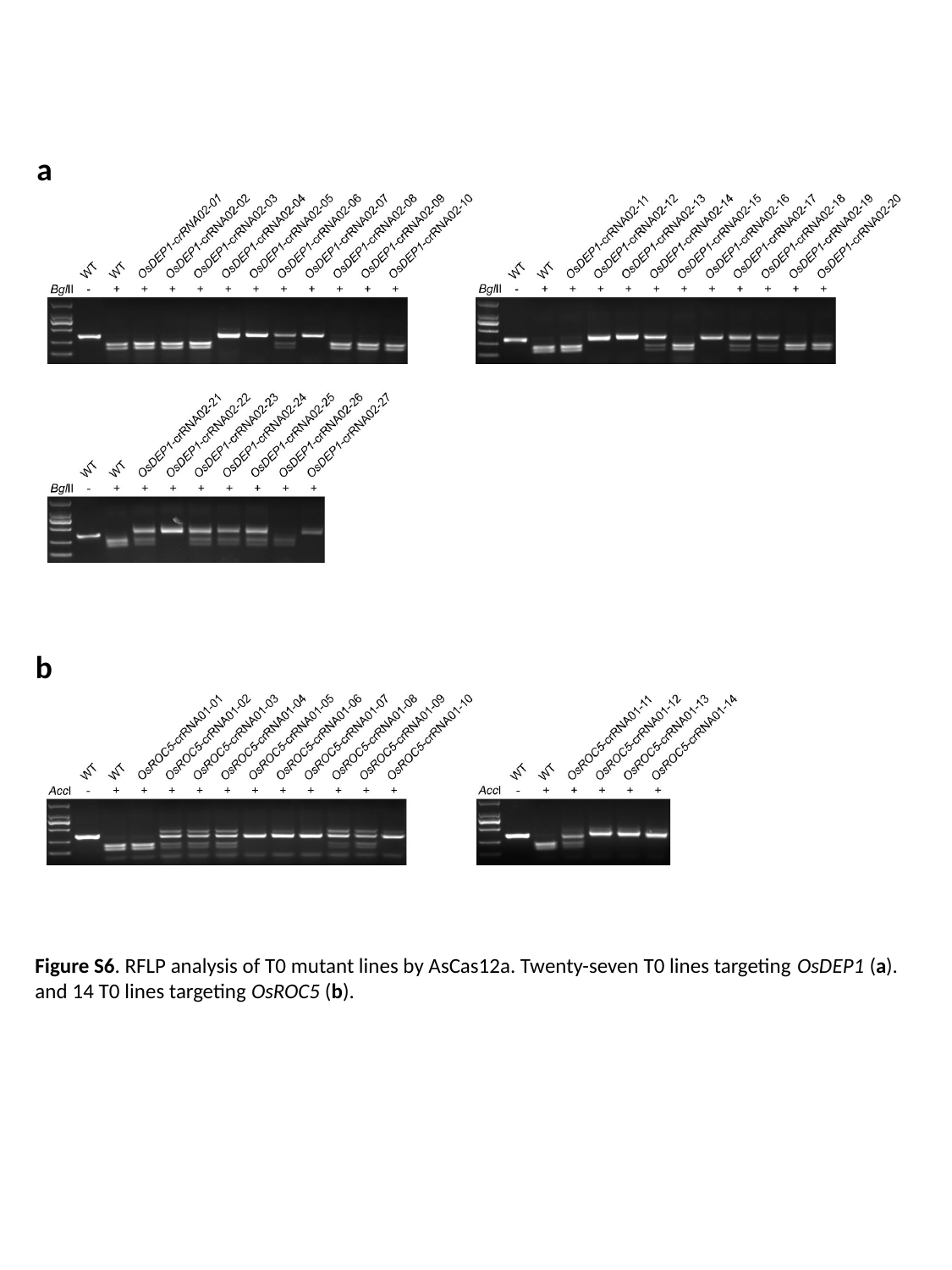

a
b
Figure S6. RFLP analysis of T0 mutant lines by AsCas12a. Twenty-seven T0 lines targeting OsDEP1 (a). and 14 T0 lines targeting OsROC5 (b).

## Slide 7
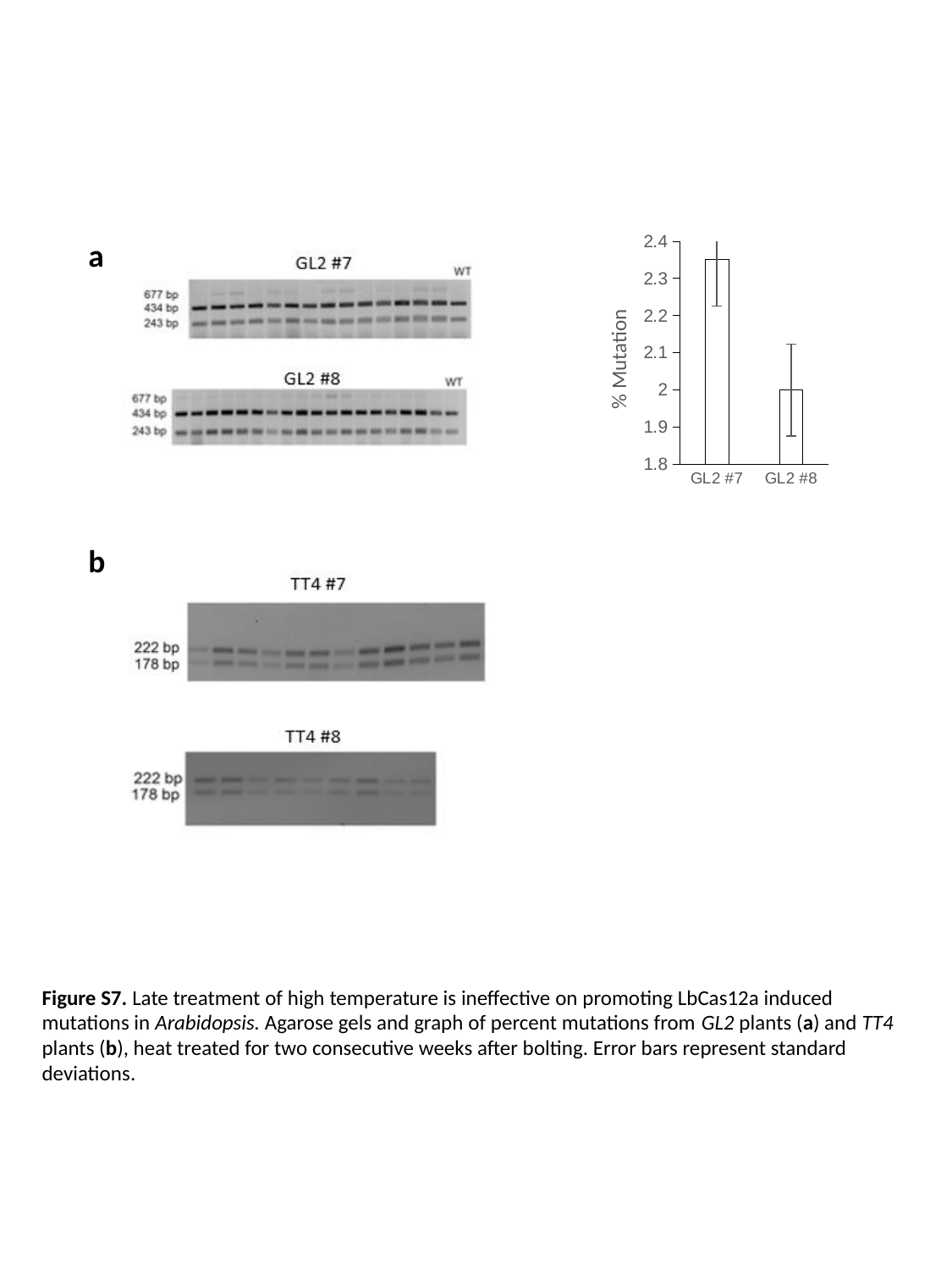

### Chart
| Category | |
|---|---|
| GL2 #7 | 2.35 |
| GL2 #8 | 2.0 |a
b
Figure S7. Late treatment of high temperature is ineffective on promoting LbCas12a induced mutations in Arabidopsis. Agarose gels and graph of percent mutations from GL2 plants (a) and TT4 plants (b), heat treated for two consecutive weeks after bolting. Error bars represent standard deviations.

## Slide 8
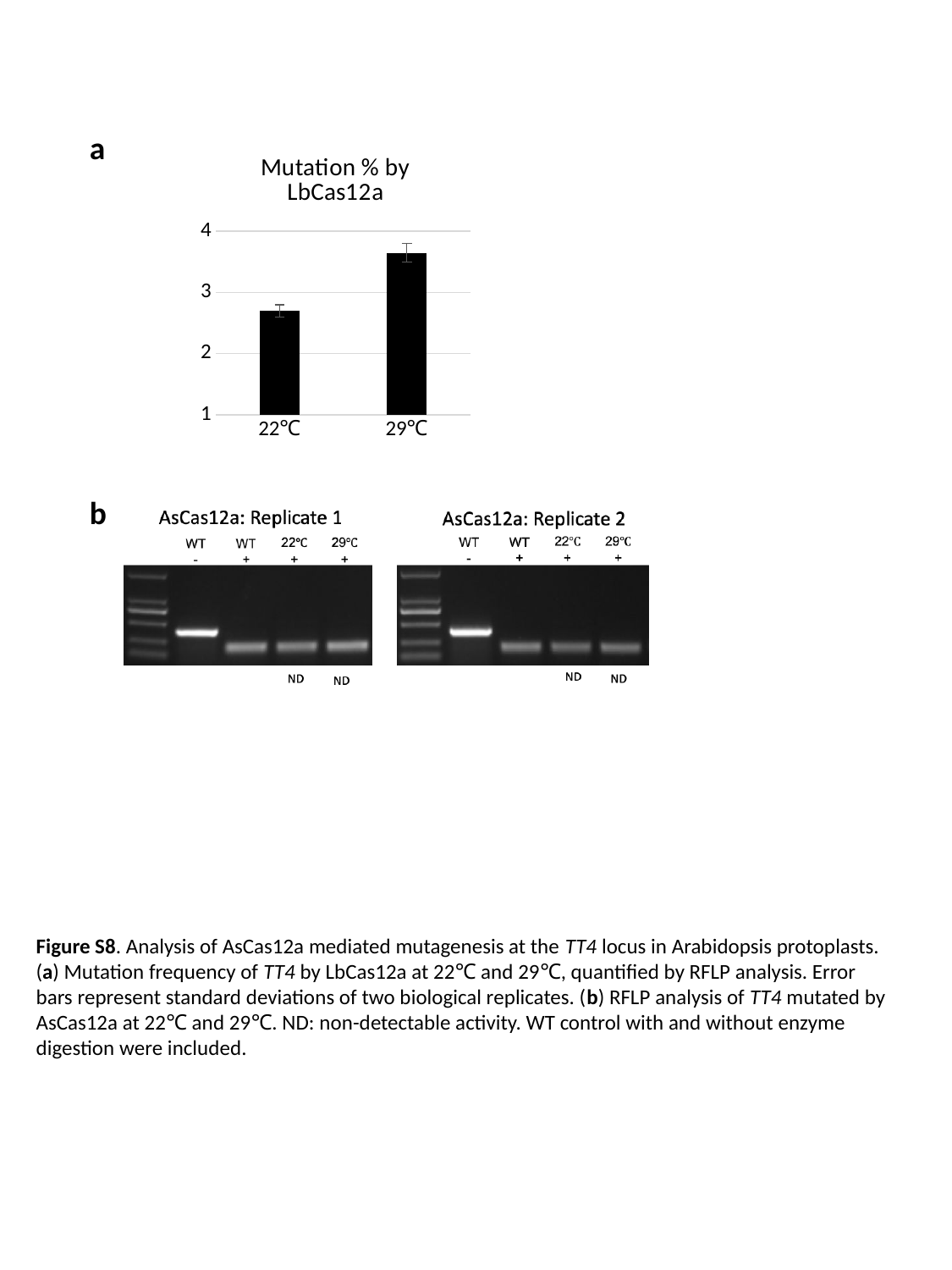

a
### Chart: Mutation % by LbCas12a
| Category | Mutation % |
|---|---|
| 22℃ | 2.7 |
| 29℃ | 3.65 |b
Figure S8. Analysis of AsCas12a mediated mutagenesis at the TT4 locus in Arabidopsis protoplasts. (a) Mutation frequency of TT4 by LbCas12a at 22℃ and 29℃, quantified by RFLP analysis. Error bars represent standard deviations of two biological replicates. (b) RFLP analysis of TT4 mutated by AsCas12a at 22℃ and 29℃. ND: non-detectable activity. WT control with and without enzyme digestion were included.

## Slide 9
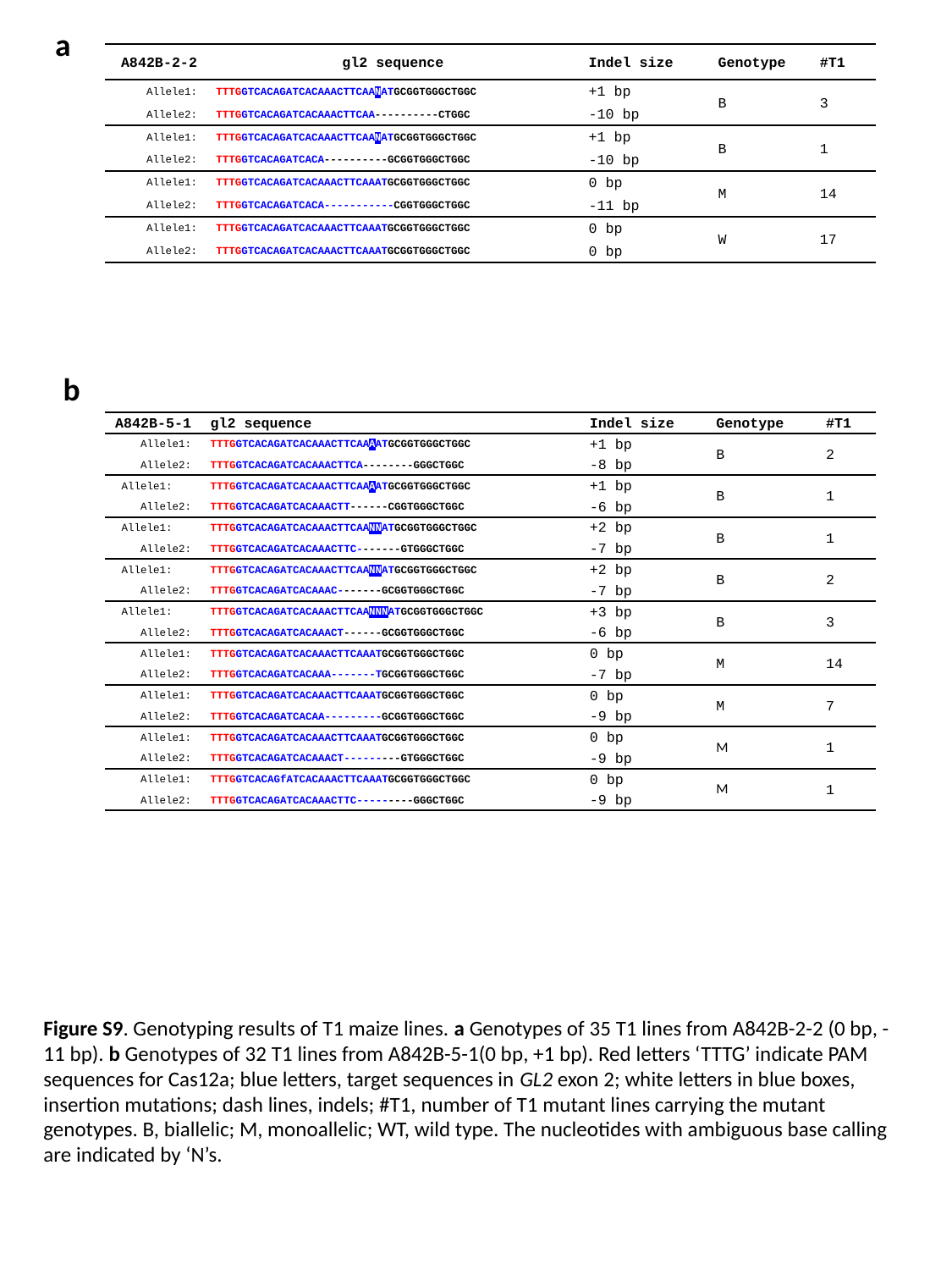

a
| A842B-2-2 | gl2 sequence | Indel size | Genotype | #T1 |
| --- | --- | --- | --- | --- |
| Allele1: | TTTGGTCACAGATCACAAACTTCAANATGCGGTGGGCTGGC | +1 bp | B | 3 |
| Allele2: | TTTGGTCACAGATCACAAACTTCAA----------CTGGC | -10 bp | | |
| Allele1: | TTTGGTCACAGATCACAAACTTCAANATGCGGTGGGCTGGC | +1 bp | B | 1 |
| Allele2: | TTTGGTCACAGATCACA----------GCGGTGGGCTGGC | -10 bp | | |
| Allele1: | TTTGGTCACAGATCACAAACTTCAAATGCGGTGGGCTGGC | 0 bp | M | 14 |
| Allele2: | TTTGGTCACAGATCACA-----------CGGTGGGCTGGC | -11 bp | | |
| Allele1: | TTTGGTCACAGATCACAAACTTCAAATGCGGTGGGCTGGC | 0 bp | W | 17 |
| Allele2: | TTTGGTCACAGATCACAAACTTCAAATGCGGTGGGCTGGC | 0 bp | | |
b
| A842B-5-1 | gl2 sequence | Indel size | Genotype | #T1 |
| --- | --- | --- | --- | --- |
| Allele1: | TTTGGTCACAGATCACAAACTTCAAAATGCGGTGGGCTGGC | +1 bp | B | 2 |
| Allele2: | TTTGGTCACAGATCACAAACTTCA--------GGGCTGGC | -8 bp | | |
| Allele1: | TTTGGTCACAGATCACAAACTTCAAAATGCGGTGGGCTGGC | +1 bp | B | 1 |
| Allele2: | TTTGGTCACAGATCACAAACTT------CGGTGGGCTGGC | -6 bp | | |
| Allele1: | TTTGGTCACAGATCACAAACTTCAANNATGCGGTGGGCTGGC | +2 bp | B | 1 |
| Allele2: | TTTGGTCACAGATCACAAACTTC-------GTGGGCTGGC | -7 bp | | |
| Allele1: | TTTGGTCACAGATCACAAACTTCAANNATGCGGTGGGCTGGC | +2 bp | B | 2 |
| Allele2: | TTTGGTCACAGATCACAAAC-------GCGGTGGGCTGGC | -7 bp | | |
| Allele1: | TTTGGTCACAGATCACAAACTTCAANNNATGCGGTGGGCTGGC | +3 bp | B | 3 |
| Allele2: | TTTGGTCACAGATCACAAACT------GCGGTGGGCTGGC | -6 bp | | |
| Allele1: | TTTGGTCACAGATCACAAACTTCAAATGCGGTGGGCTGGC | 0 bp | M | 14 |
| Allele2: | TTTGGTCACAGATCACAAA-------TGCGGTGGGCTGGC | -7 bp | | |
| Allele1: | TTTGGTCACAGATCACAAACTTCAAATGCGGTGGGCTGGC | 0 bp | M | 7 |
| Allele2: | TTTGGTCACAGATCACAA---------GCGGTGGGCTGGC | -9 bp | | |
| Allele1: | TTTGGTCACAGATCACAAACTTCAAATGCGGTGGGCTGGC | 0 bp | M | 1 |
| Allele2: | TTTGGTCACAGATCACAAACT---------GTGGGCTGGC | -9 bp | | |
| Allele1: | TTTGGTCACAGfATCACAAACTTCAAATGCGGTGGGCTGGC | 0 bp | M | 1 |
| Allele2: | TTTGGTCACAGATCACAAACTTC---------GGGCTGGC | -9 bp | | |
Figure S9. Genotyping results of T1 maize lines. a Genotypes of 35 T1 lines from A842B-2-2 (0 bp, -11 bp). b Genotypes of 32 T1 lines from A842B-5-1(0 bp, +1 bp). Red letters ‘TTTG’ indicate PAM sequences for Cas12a; blue letters, target sequences in GL2 exon 2; white letters in blue boxes, insertion mutations; dash lines, indels; #T1, number of T1 mutant lines carrying the mutant genotypes. B, biallelic; M, monoallelic; WT, wild type. The nucleotides with ambiguous base calling are indicated by ‘N’s.

## Slide 10
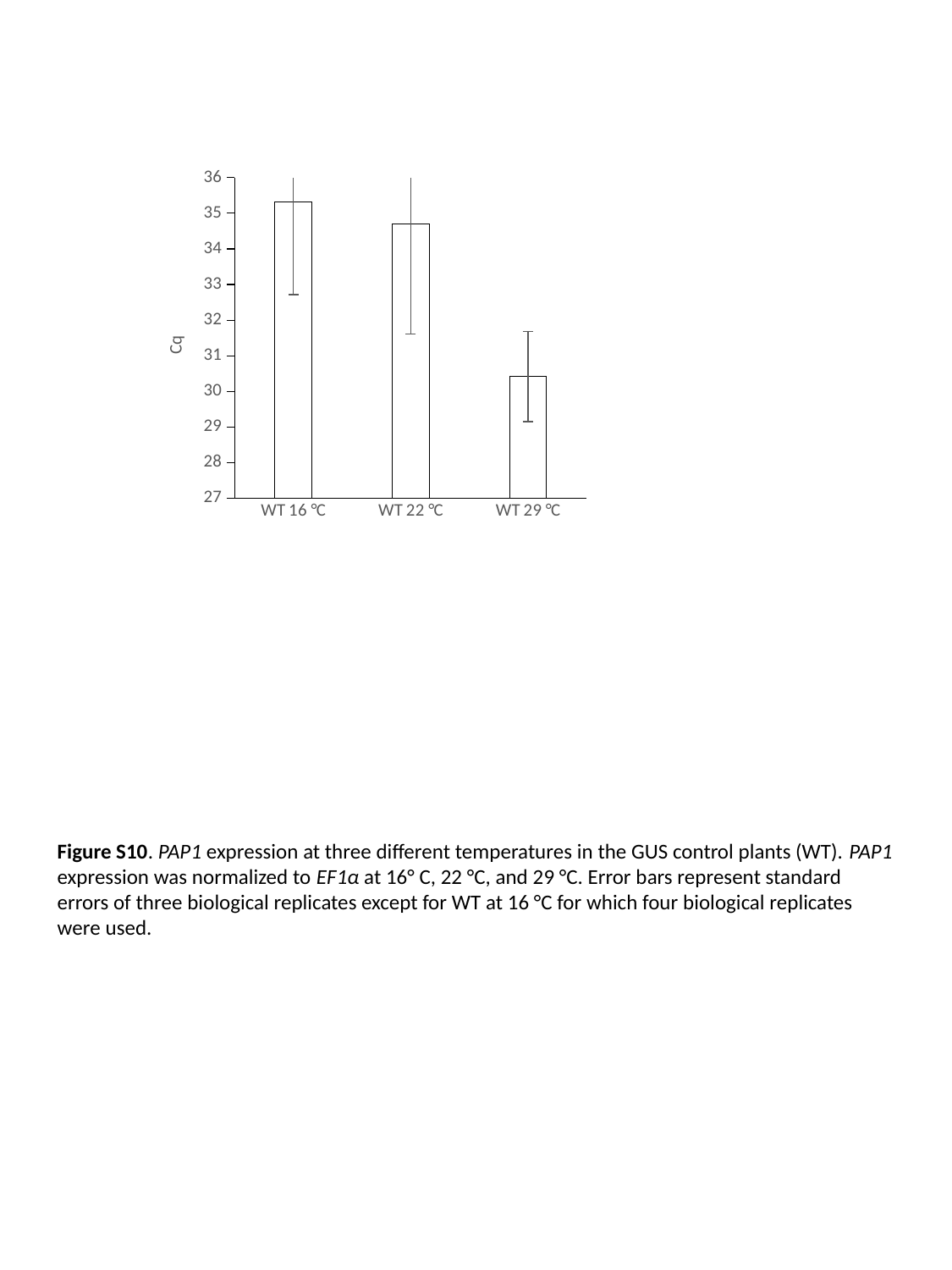

### Chart
| Category | avg |
|---|---|
| WT 16 °C | 35.31053210003 |
| WT 22 °C | 34.70185779168281 |
| WT 29 °C | 30.417372527594754 |Figure S10. PAP1 expression at three different temperatures in the GUS control plants (WT). PAP1 expression was normalized to EF1α at 16° C, 22 °C, and 29 °C. Error bars represent standard errors of three biological replicates except for WT at 16 °C for which four biological replicates were used.

## Slide 11
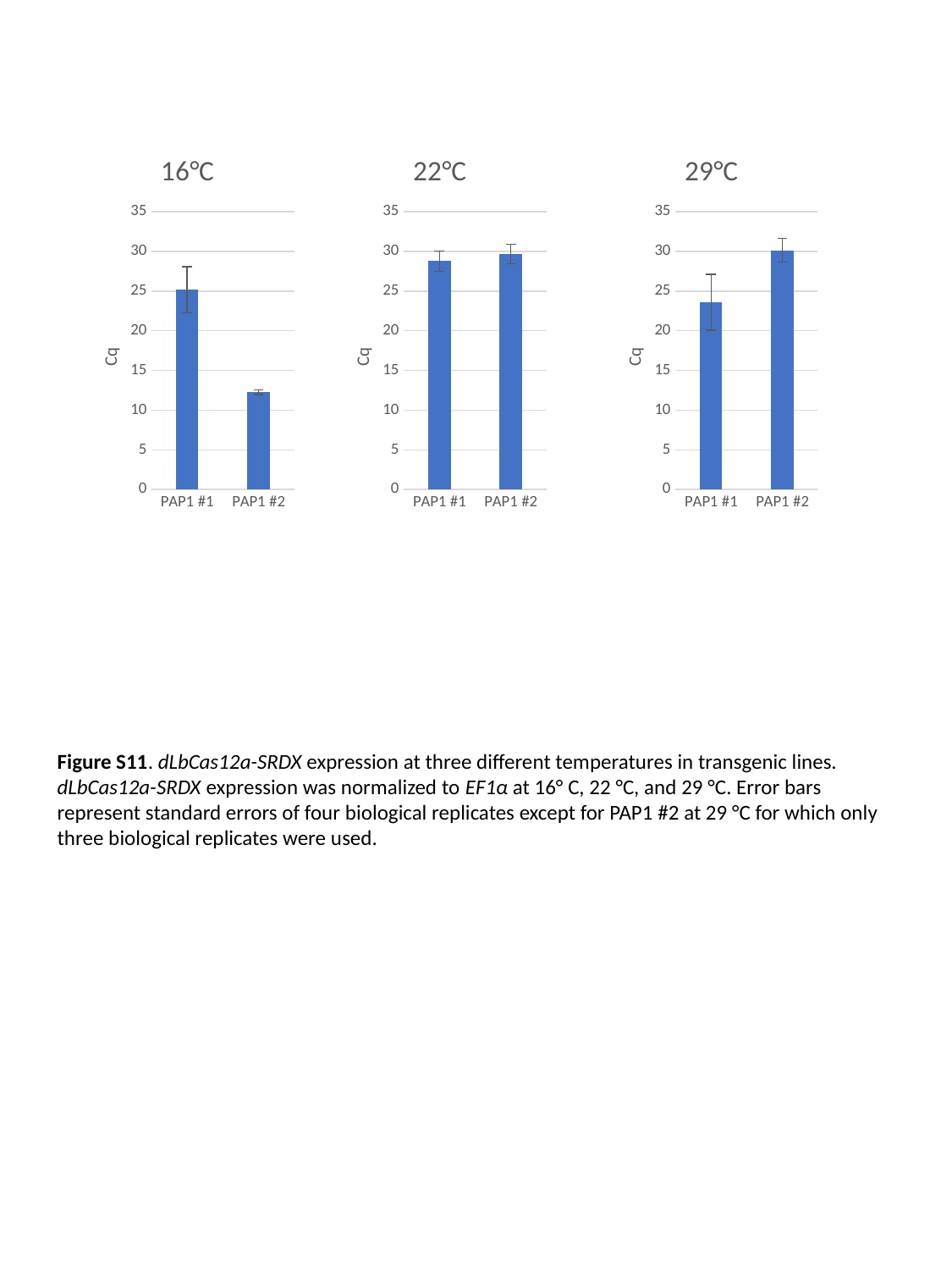

### Chart: 29°C
| Category | |
|---|---|
| PAP1 #1 | 23.611421488254845 |
| PAP1 #2 | 30.14824300495964 |
### Chart: 16°C
| Category | |
|---|---|
| PAP1 #1 | 25.166675199305054 |
| PAP1 #2 | 12.258643874294478 |
### Chart: 22°C
| Category | |
|---|---|
| PAP1 #1 | 28.777929178546707 |
| PAP1 #2 | 29.672527762931324 |Figure S11. dLbCas12a-SRDX expression at three different temperatures in transgenic lines. dLbCas12a-SRDX expression was normalized to EF1α at 16° C, 22 °C, and 29 °C. Error bars represent standard errors of four biological replicates except for PAP1 #2 at 29 °C for which only three biological replicates were used.

## Slide 12
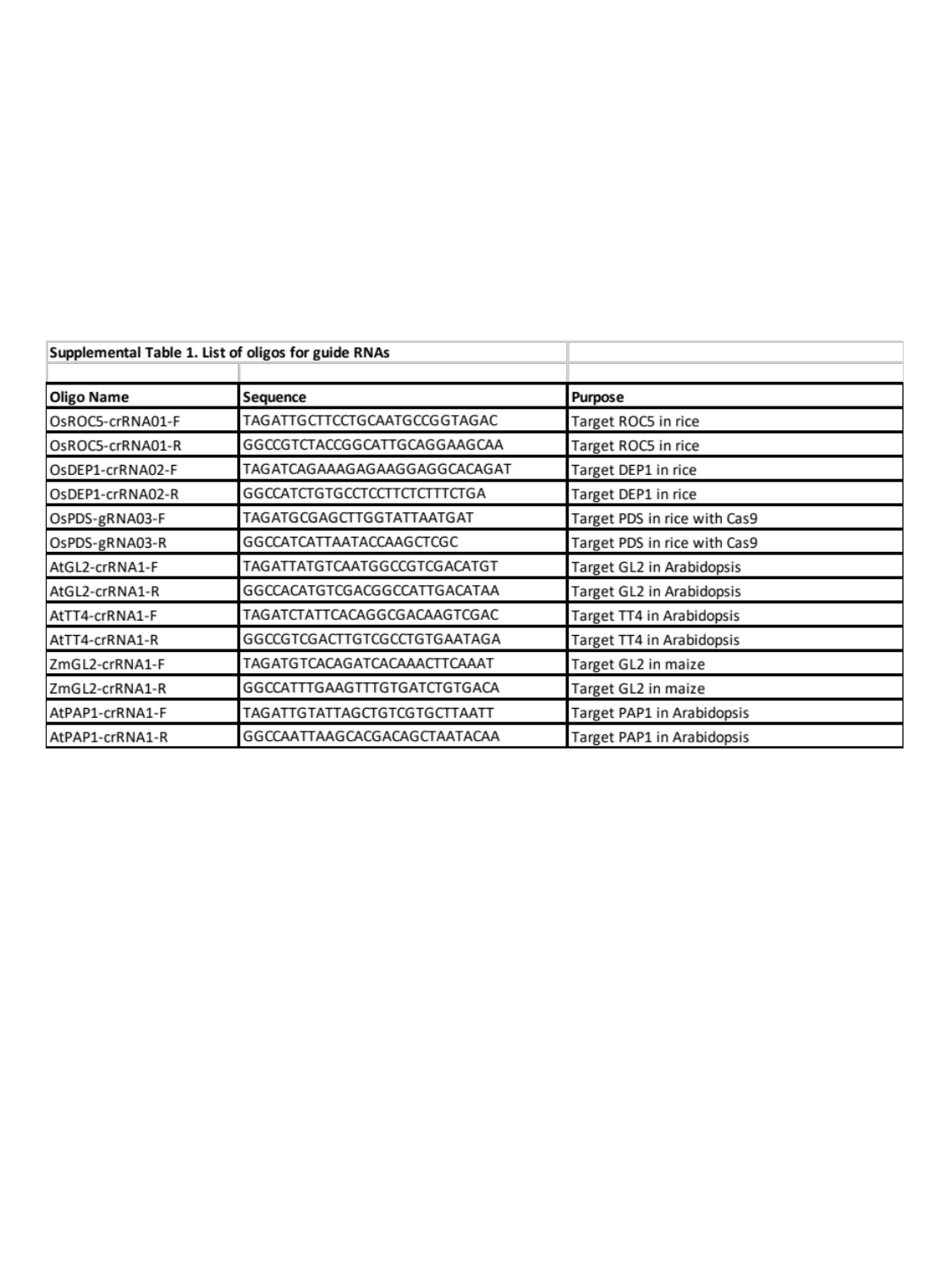

Supplement: Supplementary file 1 — Figure S1.RFLP analysis of nuclease activities of three Cas12a (Cpf1) nucleases in rice protoplasts under different temperatures. Figure S2. RFLP analysis of SpCas9 activity in rice protoplasts under different temperatures. Figure S3. Measurement of rice protoplast transfection efficiencies using GFP construct at different temperatures. Figure S4. Three Cas12a nucleases generate slightly distinct mutation profiles at OsROC5 site which are unaffected by temperature. Figure S5. Mutation profiles by SpCas9 are largely unaffected by temperature. Figure S6. RFLP analysis of T0 mutant lines by AsCas12a. Figure S7. Late treatment of high temperature is ineffective on promoting LbCas12a-induced mutations in Arabidopsis. Figure S8. Analysis of AsCas12a-mediated mutagenesis at the TT4 locus in Arabidopsis protoplasts. Figure S9. Genotyping results of T1 maize lines. Figure S10. PAP1 expression at three different temperatures in the GUS control plants (WT). Figure S11. dCas12a-SRDX expression at three different temperatures in transgenic lines. Table S1. Guide RNA oligos. (PPTX 10187 kb) [file 12915_2019_629_MOESM1_ESM.pptx]
